# Cyano-Polycyclic Aromatic Hydrocarbon Interstellar Candidates: Laboratory Identification, Equilibrium Structure and Astronomical Search of Cyanobiphenylene

**DOI:** 10.1021/acs.jpclett.4c01500

**Published:** 2024-07-12

**Authors:** Carlos Cabezas, Jesús Janeiro, Dolores Pérez, Wenqin Li, Marcelino Agúndez, Amanda L. Steber, Enrique Guitián, Jean Demaison, Cristóbal Pérez, José Cernicharo, Alberto Lesarri

**Affiliations:** † Instituto de Física Fundamental, CSIC, C/Serrano 123, 28006 Madrid, Spain; ‡ Centro Singular de Investigación en Química Biolóxica e Materiais Moleculares (CiQUS) and Departamento de Química Orgánica, Universidade de Santiago de Compostela, 15782 Santiago de Compostela, Spain; § Departamento de Química Física y Química Inorgánica, Facultad de Ciencias. - I.U. CINQUIMA, Universidad de Valladolid, 47011 Valladolid, Spain; ○ Physique des Lasers, Atomes et Molécules, Université de Lille, Bât. P5, 59655 Villeneuve d’Ascq, France

## Abstract

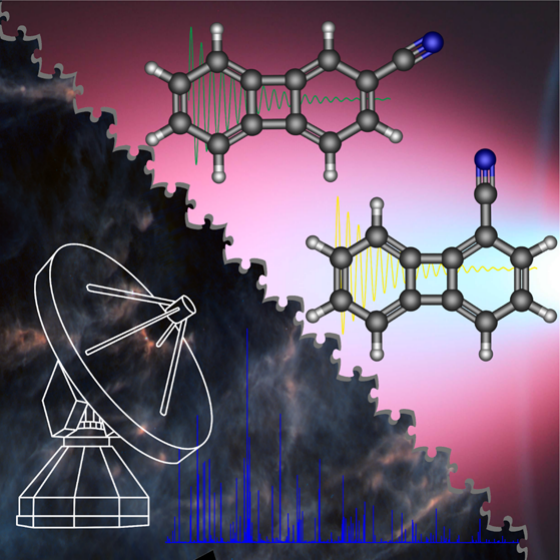

The interplay between laboratory rotational spectroscopy and radio astronomical observations provides the most effective procedure for identifying molecules in the interstellar medium (ISM). Following the recent interstellar detections of several Polycyclic Aromatic Hydrocarbons (PAHs) and cyano derivatives in the dense molecular cloud TMC-1, it is reasonable to consider searching for other cyano-PAHs in this astronomical source. We present a rotational spectroscopy investigation of the two cyano derivatives of the PAH biphenylene, a plausible reaction product of interstellar benzyne. The rotational spectrum provided molecular parameters for the parent species and 14 monosubstituted isotopologues for each isomer. An accurate equilibrium structure was determined for both isomers using Watson’s mass-dependence method (*r*_m_^(2)^), offering information on its uncommon ring union. Astronomical searches for the cyanobiphenylene isomers have been undertaken in TMC-1, using the QUIJOTE line survey. No lines of any isomer were found in this astronomical source, but the experimental data will serve to enable future searches for these species in the ISM.

In the mid-1980s, the PAH hypothesis was born.^1,2^ It proposed that the unidentified infrared emission features found in the interstellar medium (ISM) are carried out by large Polycyclic Aromatic Hydrocarbon (PAHs) molecules. These species have an abundance of 5–10% of the elemental interstellar carbon and constitute the most abundant organic molecules in space. Over the last four decades, the PAH hypothesis has been largely accepted. However, it has also received criticism^3^ based on the non-detection of individual PAH molecules. The astronomical identification of about 90% of all the known molecules in space has been achieved by their pure rotational signatures and radio astronomy.^4^ The discovery of new molecules usually results from a synergic work within the fields of astronomy, laboratory spectroscopy, and quantum chemistry. The unambiguous astronomical identification is very difficult for PAHs due to their high symmetry and negligible or null electric dipole moments. In fact, the first pure PAH, indene (C_9_H_8_), was detected in the cold pre-stellar core Taurus Molecular Cloud (TMC-1) only in 2021.^5,6^ In the same astronomical source, the cyano derivatives of cyclopentadiene (C_5_H_5_CN),^7,8^ benzene (C_6_H_5_CN),^9^ indene (C_9_H_7_CN)^10^, and naphthalene (C_10_H_7_CN)^11^ have been detected recently. Additional observations of ethynyl derivatives for cyclopentadiene (C_5_H_5_CCH)^12^ and benzene (C_6_H_5_CCH)^13^ also confirm the view that aromatic rings are abundant in cold clouds.^14^ The nitrile group in these cyano derivatives largely increases their dipole moments with respect to their pure cyclic counterparts and makes their rotational transitions much easier to observe. Sita *et al*.^10^ posit that cyano derivatives of PAHs can indeed be used as observational proxies to refine the astrophysical models for their hydrocarbon counterparts. Hence, the astronomical detection of polar derivatives of the target PAH molecules will certainly contribute to improve our understanding on the chemical inventories of PAH molecules.

Biphenylene (C_12_H_8_, hereafter BP) is one of the most interesting unsaturated hydrocarbons. It has a unique 6-4-6 arene assembly of *D_2h_* symmetry in which two benzene rings are joined by two bridging bonds instead of a ring fusion. X-ray and gas-phase electron diffraction studies have confirmed important bond length alternations (ca. 1.37-1.42 Å) and long bridge bonds (ca. 1.52 Å).^15,16^ According to the Hückel 4*n* π-electron rule, BP is an antiaromatic molecule, normally characterized by enhanced reactivity and photophysical properties. Electron delocalization has been observed in the center ring,^17^ but the large separation between the two benzyne units reduces the interaction between the rings. Therefore, the molecule can be considered borderline between aromatic and antiaromatic behavior.^18−20^ In spite of its partial antiaromaticity, BP is considerably stable in comparison to other antiaromatic compounds and shows particular chemical properties. Thus, it has attracted much attention from fields like catalysis or material science due to its reactivity with transition metal complexes^21^ and its capability to form carbon nanostructures with valuable mechanical, electronic, and transport properties.^22^

The BP molecule can also be viewed as the result of the dimerization of benzyne (C_6_H_4_), which has been detected very recently in TMC-1^23^ thanks to the high sensitivity of the QUIJOTE^23,24^ radioastronomical survey. Quantum chemical calculations (see Experimental Methods) show that the dimerization reaction of benzyne produces BP as major product and without any reaction barrier. Hence, it is straightforward to consider that BP could also be present in TMC-1, where benzyne and other PAHs have been detected.^5−11^ However, the high symmetry of BP does not permit a permanent electric dipole moment, so it cannot be detected by rotational spectroscopy. In this scenario, the cyano derivatives of BP, named 1- and 2-cyanobiphenylene (1-CNBP and 2-CNBP, C_13_H_7_N, see Figure 1), are good candidates to be observed in TMC-1. To enable the interstellar searches for 1-CNBP and 2-CNBP, their pure rotational spectra need to be investigated in the laboratory. To the best of our knowledge, no spectral information exists in the literature for any of these cyano compounds which, in fact, are not commercially available. We report here a high-resolution spectroscopic investigation of 1-CNBP and 2-CNBP using broadband chirped-pulse Fourier transform microwave (CP-FTMW) spectroscopy.^25,26^ Accurate spectroscopic parameters were derived from the analysis of the experimental spectra in the cm-wave region (2–14 GHz), allowing for frequency predictions to perform astronomical searches. The sensitive rotational observations further permitted the derivation of near-equilibrium structures for 1-CNBP and 2-CNBP, which are unprecedented considering the relative large molecular size of these molecules.

**Figure 1:**
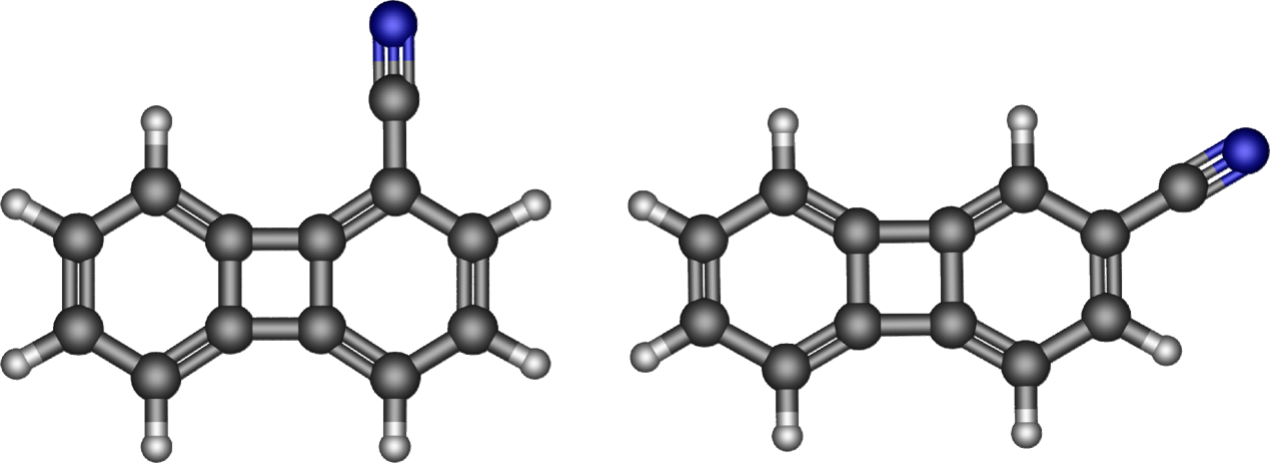
Molecular structures of the cyanobiphenylenes 1-CNBP (left) and 2-CNBP (right).

The molecules of 1-CNBP and 2-CNBP were synthesized for this work by the reaction of the corresponding bromobiphenilenes^27^ (1-bromo and 2-bromobiphenylene, or 1-BBP and 2-BBP) using cuprous cyanide in refluxing dimethylformamide (DMF), as described in the Experimental Methods and the Supporting Information. The solid samples of 1-CNBP and 2-CNBP were later vaporized by thermal heating (∼100°C) and prepared in a jet expansion to be probed in the microwave spectrometer (see Experimental Methods). The rotational spectra for both isomers, shown in Figures 2 and S1, show very intense rotational lines and a line profile corresponding to a rotational temperature of ca. 2 K. The spectral identification of both species was assisted by quantum chemical predictions using density functional theory and MP2 computations. We summarize in Table 1 the MP2/cc-pVTZ results.^28,29^ As illustrated in Figure 2, each rotational transition is split into several hyperfine components because of the nuclear quadrupole coupling effects produced by the presence of a ^14^N nucleus.^30^ The frequencies for each of the resolved hyperfine components were fit to experimental accuracy (10 kHz) using a Watson’s *S*-reduced semirigid Hamiltonian (*I*^r^ representation)^31^ with Pickett’s CALPGM programs.^32^ A total of 724 hyperfine components with quantum numbers 1≤ *J* ≤16 and 0≤ *K_a_* ≤7 were measured and fit for 1-CNBP, while for 2-CNBP the number of fit lines rises to 655 with quantum numbers 0≤ *J* ≤20 and 0≤ *K_a_* ≤5. A list of lines observed for each isomer is given in the Supporting Information. The fits reproduce very well the measured frequencies since the root mean square (RMS) deviations are smaller than the accuracy of the measurements, 8.2 and 9.7 kHz for 1-CNBP and 2-CNBP, respectively. This analysis provided experimental accurate values for the rotational constants, centrifugal distortion constants and three elements of the ^14^N nuclear quadrupole coupling tensor of both molecules, shown in Table 1. The high sensitivity of the experiment additionally allowed the observation of the spectra of all 13 monosubstituted ^13^C- and ^15^N-isotopologues in natural abundance (∼1.1% and 0.4%, respectively) for each structural isomer. The minor isotopologues were analyzed similarly to the parent species and their derived parameters together with the observed frequencies are given in the Supporting Information. The molecular parameters for 1-CNBP and 2-CNBP in Table 1 represent the first experimental gas phase information available for these species. As it can be observed, the title compounds are quite rigid, and only two centrifugal constants were required in the fit. The rigidity of the molecule also results in a very good agreement (relative differences <0.4%) of the rotational constants with those predicted from quantum chemical calculations.

**Figure 2:**
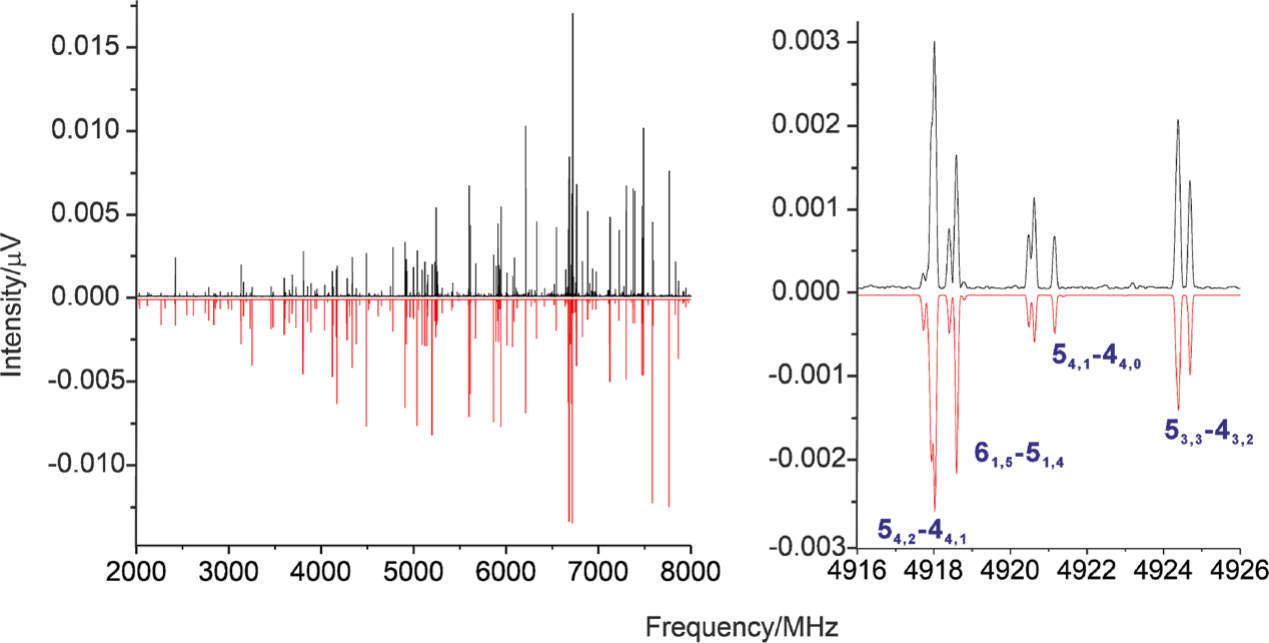
Broadband microwave spectrum of 1-CNBP (1.4 million averages) in the region 2–8 GHz. The experimental trace is represented by the positive black trace. The negative red trace is a simulation (at 2 K) using the fitted rotational parameters of Table 1. The right inset displays several rotational transitions illustrating the nuclear quadrupole hyperfine pattern.

**Table 1:** Rotational Parameters for the Cyanobiphenylene Molecules 1-CNBP and 2-CNBP^a^

	1-CNBP		2-CNBP	
	Experiment	Theory	Experiment	Theory
*A*_0_ / MHz^b^	1242.06201(20)^d^	1236.0	2365.94533(54)	2356.8
*B*_0_ / MHz	575.741572(81)	574.1	385.355406(81)	384.0
*C*_0_ / MHz	393.433777(78)	392.1	331.450708(81)	330.2
*D_J_* / kHz	0.01313(61)	0.012	0.00161(20)	0.0021
*D_JK_* / kHz	[0.022]^e^	0.022	[−0.022]	–0.022
*D_K_* / kHz	0.0251(68)	0.025	0.627(58)	0.506
χ_*aa*_ / MHz	1.6171(25)	1.50	–3.4744(30)	–3.22
χ_*bb*_ / MHz	–3.5606(27)	–3.44	1.5904(36)	1.32
χ_*ab*_ / MHz	±1.98(52)^f^	±1.62	±1.73(21)^f^	±1.88
|μ_a_| / D	–	2.1	–	5.6
|μ_b_| / D	–	3.9	–	1.7
*N*^c^	724	–	655	–
σ / kHz	8.2	–	9.7	–

a The experimental values correspond to the vibrational ground-state while the predictions were done at the MP2/cc-pVTZ level (anharmonic vibrational frequencies).

b Ground-state rotational constants (*A*_0_, *B*_0_, *C*_0_), Watson’s S-reduction centrifugal distortion constants (*D_J_*, *D_JK_*, *D_K_*; *d*_1_ = *d*_2_ = 0), nuclear quadrupole coupling constants (**χ**_α,**β**_, α, **β** = *a*, *b*, *c*) and electric dipole moments (*μ_α_*, α = *a*, *b*; *μ_c_* = 0).

c Number of transitions (*N*) and rms deviation (σ) of the rotational fit.

d Values in parentheses denote 1σ errors, applied to the last digit.

e Parameters in square brackets were kept fixed in the fit.

f Only the relative sign is determined.

The abundant isotopic information permitted the determination of an equilibrium structure for the two cyanobiphenylenes. The structural calculations were conducted with Watson’s mass-dependence biparametric model (*r*_m_^(2)^).^33^ In this method the differences between the observed ground-state moments of inertia (*I*_0_^ξ^, *ξ = a,b,c*) and the equilibrium values (*I*_m_^ξ^) are modeled by two terms depending on the square root of the equilibrium moments and the atomic masses m_*i*_, according to
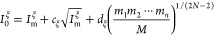
1where *c_ξ_* and *d_ξ_* are the fitting parameters for each axis. Full details of the structural calculation are given in the SI. In the final fit of Table 2 four rovibrational parameters were fitted, with the hydrogen atom parameters fixed to the predicate values in the SI (alternative fits in Tables S1 and S2). The expected accuracy of the *r*_m_^(2)^ structure is in the range of the mÅ and tenth of degree. For comparison, the MP2/cc-pVTZ prediction also in Table 2 shows differences of 1-18 mÅ and 0.1-0.3° with respect to the *r*_m_^(2)^ structure. In addition to the long separation between the phenyl rings small structural changes (<10 mÅ) are observed in the biphenylene core depending on the substitution position. The cyanobiphenylenes represent one of the largest molecules for which an equilibrium structure was determined, offering reference values for the uncommon bridged structure of the title compounds.^34^

**Table 2:**
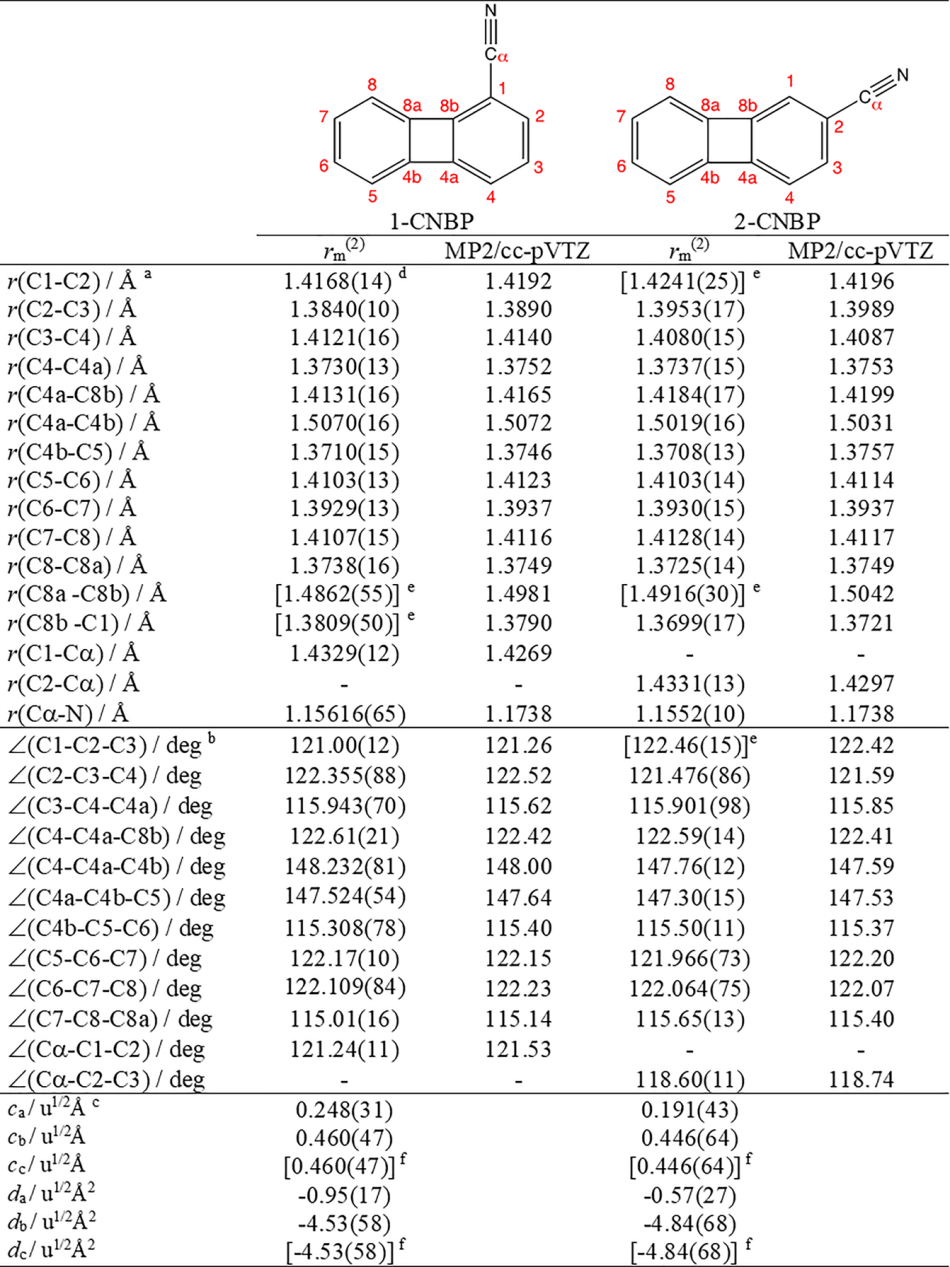
Equilibrium Structures (*r*_m_^(2)^) of 1-Cyano and 2-Cyanobiphenylene and Comparison with the *Ab Initio* Optimizations

a Bond lengths.

b Bond angles.

c Rovibrational parameters.

d Standard errors in parentheses in units of the last digit.

e Derived parameter.

f Rovibrational parameters in square brackets were fixed in the fit.

Finally, we used the experimental molecular parameters and the same Hamiltonian^32^ to obtain frequency predictions up to 50 GHz, providing a means for the 1-CNBP and 2-CNBP astronomical detection. Even though the frequency range of our laboratory observations was not too broad, the frequency extrapolation of our predictions is feasible due to the accuracy of the obtained molecular constants and the large rigidity of the two molecules. The frequency predictions for 1-CNBP and 2-CNBP allowed a search for both species in the cold dark cloud TMC-1, where they could be present considering that other large organic cycles have been detected there recently.^5−14^ We used data from QUIJOTE, a very sensitive Q band (31.0-50.3 GHz) line survey observed with the Yebes 40 m radiotelescope. Currently, the QUIJOTE data amount a total on-source telescope time of 1202 h, with the sensitivity in antenna temperature ranging between 0.08 mK at 32 GHz and 0.2 mK at 49.5 GHz. At low temperatures around 10 K, the most favorable lines for detection of 1-CNBP are *b*-type transitions with *K*_a_ > 10 at frequencies below 40 GHz. This is also the most favorable frequency range of the QUIJOTE data because of the lower noise level. In the case of 2-CNBP, the most favorable lines are a-type transitions with *K*_a_ > 10 lying also below 40 GHz. We therefore, searched for these lines in our QUIJOTE data. However, we did not detect the target rotational transitions at the current level of sensitivity. We show in Figure 3 the TMC-1 spectra around three of these favorable lines for 1-CNBP and 2-CNBP. The line parameters for these lines are shown in Table 3. Assuming a rotational temperature of 9 K, which is the gas kinetic temperature in TMC-1, a line width of 0.6 km s^–1^,^14^ and a systemic velocity of TMC-1 is 5.83 km s^–1^,^35^ also typical of TMC-1, 3σ upper limits to the beam-averaged column density of 1-CNBP and 2-CNBP in TMC-1 of 9.6 × 10^10^ cm^–2^ and 5.2 × 10^11^ cm^–2^, respectively, have been derived (see details in the Supporting information). These upper limits are 1-2 orders of magnitude lower than the abundances derived or estimated for the two-ring molecules indene (C_9_H_8_) and naphthalene (C_10_H_8_),^36^ but comparable to the abundance estimated for benzyne (C_6_H_4_).^23^

**Figure 3:**
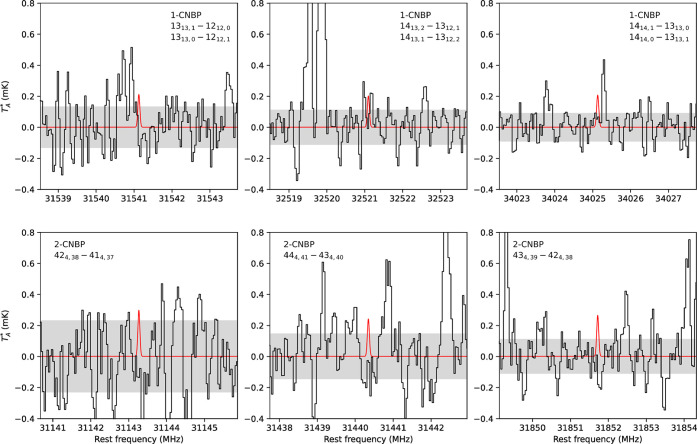
Spectra of TMC-1 from the QUIJOTE survey at the frequencies of some of the most intense predicted lines of 1-CNBP and 2-CNBP. Quantum numbers for each rotational transition are indicated in each panel. We show as a red trace the synthetic line profiles calculated for the column density upper limits given in the text. The 3σ detection limits are indicated in grey. As observed, none of these species are detected in TMC-1 at the current level of sensitivity of the QUIJOTE data. Line parameters are shown in Table 3.

**Table 3:** Line Parameters of the Rotational Transitions for 1-CNBP and 2-CNBP Shown in Figure 1

Transition	Frequency (MHz)	*E_up_* (K)^a^	*A* (s^–1^)^b^	*g_up_*^a^
1-CNBP				
13_13,1_-12_12,0_	31541.130	10.4	2.56×10^–6^	27
13_13,0_-12_12,1_	31541.130	10.4	2.56×10^–6^	27
14_13,2_-13_12,1_	32521.097	11.0	2.59×10^–6^	29
14_13,1_-13_12,2_	32521.097	11.0	2.59×10^–6^	29
14_14,1_-13_13,0_	34025.132	12.0	3.23×10^–6^	29
14_14,0_-13_13,1_	34025.132	12.0	3.23×10^–6^	29
2-CNBP				
42_4,38_-41_4,37_	31143.272	33.3	5.40×10^–6^	85
44_4,41_-43_4,40_	31440.348	35.7	5.55×10^–6^	89
43_4,39_-42_4,38_	31851.719	34.8	5.78×10^–6^	87

a *E_up_* and *g_up_* are the energy and statistical weight, respectively, of the upper level.

b Einstein’s coefficient.

In conclusion, the present investigation represents a comprehensive chemical, spectroscopic, and structural investigation of 1-cyano and 2-cyanobiphenylenes, not attempted before in the gas phase. The spectral study, comprising the determination of rotational parameters for 15 different isotopologues for each species, simultaneously provided empirical evidence for the determination of the equilibrium structure and the radioastronomical search in the Q band. The negative detection in TMC-1 led to an upper limit in this interstellar source considerably lower than other PAHs. This information could be used for radioastronomical models of abundance of the title compounds. Finally, we expect that the search for undetected PAHs in space will be boosted in the near future by the confluence of highly sensitive radiotelescope surveys and laboratory experiments using chirped-pulse broadband microwave spectroscopy, which combines wide broadband operation and excellent sensitivity and dynamical range.

## Experimental Methods

### Sample Synthesis

All reactions were carried out under argon using oven-dried glassware. Anhydrous THF, CH_2_Cl_2_ and DMF were taken from a MBraun SPS-800 Solvent Purification System. *n*-BuLi was titrated by using the diphenyl acetic acid method. Commercial reagents were purchased from ABCR GmbH, Sigma-Aldrich or Fluorochem, and were used without further purification. 2,2′,6-Tribromobiphenyl was prepared following a previously published procedure. TLC was performed on Merck silica gel 60 F254 and chromatograms were visualized with UV light (254 and 360 nm). Column chromatography was performed on Merck silica gel 60 (ASTM 230-400 mesh). Centrifugation was performed in a Hettich EBA21 centrifuge. ^1^H and ^13^C NMR spectra were recorded at 300 and 75 MHz (Varian Mercury-300 instrument), 400 and 101 MHz (Varian Inova 400) or 500 and 125 MHz (Varian Inova 500) respectively. Low resolution mass spectra (EI) were obtained at 70 eV on a HP-5988A instrument, while high-resolution mass spectra (HRMS) were obtained on a Micromass Autospec spectrometer. Atmospheric pressure chemical ionization (APCI) HRMS were obtained on a Bruker MicroTOF, using either Direct Inlet Probe (DIP) or Flow Injection Analysis (FIA) for sample introduction. UV-Vis and fluorescence spectra were obtained on a Jasco V-630 and on a Fluoromax-2 spectrophotometers, respectively.

### Quantum Chemical Calculations

Prior to the experimental work, we performed quantum chemical calculations to obtain the 1-CNBP and 2-CNBP optimized structures at the Møller–Plesset (MP2)^28^ perturbation theory in the frozen core approximation method together with the Dunning’s correlation consistent basis sets with polarized core–valence triple-ζ (cc-pVTZ).^29^ Rotational constants, nuclear quadrupole coupling constants and electric dipole moment components were derived from these calculations. Anharmonic vibrational frequencies were computed using the B3LYP hybrid density functional^37^ with the cc-pVTZ basis set to estimate the centrifugal distortion constants and rovibrational contributions. On the other hand, the potential energy surface for the dimerization reaction of benzyne to form BP molecule was explored y quantum chemical calculations at B3LYP/cc-pVTZ level of calculation. We found that the dimerization reaction proceeds directly to the formation of BP through a barrierless mechanism. No stable pre-reactive complex or transition state could be located despite a detailed analysis of the potential energy surface. Indeed, all the geometry optimizations with different starting points led to BP molecule. All the calculations were performed using the Gaussian16 program package.^38^

### Rotational Spectroscopy

The 1-CNBP and 2-CNBP structural isomers were studied in independent experiments using a broadband chirped-pulsed Fourier-transform microwave (CP- FTMW) spectrometer at the Universidad de Valladolid working in the 2-14 GHz frequency region.^25,39^ The solid samples were vaporized at moderate temperatures (ca. 100°C) in a solenoid-driven pulsed injector and diluted with an inert carrier gas (neon, 3 bar). Expansion of the gas mixture into an evacuated chamber generates molecular jet pulses (typ. 800 μs). The data was collected in a series of different measurements. In the first one, the molecules of each gas pulse were polarized with a series of 8 microwave pulses with a duration of 4 μs spanning 2-8 GHz. The measurement was performed at a repetition rate of 5 Hz, giving an effective repetition rate of 40 Hz. The 8-14 GHz regime was divided into three separate measurements, 2 GHz each, in which each gas pulse was probed with 5 microwave pulses. The operating repetition rate of the instrument was 1 Hz, giving an effective repetition rate of 5 Hz. The chirp pulses were generated with an arbitrary waveform generator (Tektronix AWG 70002A), amplified by 300 W with a traveling-wave tube amplifier (TWT) in the case of the 2-8 GHz measurement and by 20 W with a solid-state MW amplifier in the case of the higher frequency measurements, and broadcasted perpendicular to the propagation of the jet expansion through a horn antenna. The use of a Kaiser-Bessel apodization window resulted in a typical full width at half-maximum (FWHM) line width of ca. 100 kHz. The accuracy of the frequency measurements is better than 10 kHz. All frequency components are referenced to a Rb standard.

## Supporting Information Available

Additional experimental, computational and structural methods; details of the upper limit of column density estimation; supplementary Figures S1–S5 and Tables S1–S7, including synthesis and characterization data and computational results (PDF)
List of the observed rotational transitions and experimental molecular constants for the parent and isotopologues of 1-CNBP and 2-CNBP (ZIP)
