# Supplementary material for: Cyano-Polycyclic Aromatic Hydrocarbon Interstellar Candidates: Laboratory Identification, Equilibrium Structure and Astronomical Search of Cyanobiphenylene
Source: J Phys Chem Lett. 2024 Jul 12;15(29):7411–8. doi: 10.1021/acs.jpclett.4c01500 (PMC11284842; doi:10.1021/acs.jpclett.4c01500)
Supplement: Supplementary file 1 — jz4c01500_si_001.pdf [file jz4c01500_si_001.pdf]

Cyano-Polycyclic Aromatic Hydrocarbon Interstellar Candidates: Laboratory Identification, Equilibrium Structure and Astronomical Search of Cyanobiphenylene

Supporting Information

Carlos Cabezas†, Jesús Janeiro‡, Dolores Pérez‡, Wenqin Li§, Marcelino Agúndez†, Amanda L. Steber§, Enrique Guitián‡, Jean Demaison°, Cristóbal Pérez§, José Cernicharo† and Alberto Lesarri§**

† Instituto de Física Fundamental, CSIC, C/ Serrano 123, 28006 Madrid, Spain
‡ Centro Singular de Investigación en Química Biolóxica e Materiais Moleculares (CiQUS) and Departamento de Química Orgánica, Universidade de Santiago de Compostela, 15782 Santiago de Compostela, Spain
§ Departamento de Química Física y Química Inorgánica, Facultad de Ciencias. - I.U. CINQUIMA, Universidad de Valladolid, 47011 Valladolid, Spain
° Physique des Lasers, Atomes et Molécules, Université de Lille, Bât. P5, 59655 Villeneuve d'Ascq cedex, France

Experimental Chemical Synthesis Methods

1. Synthesis of 1-cyanobiphenylene (1-CNBP) and 2-cyanobiphenylene (2-CNBP): experimental details and spectroscopic data

1.1. Experimental procedures and characterization data.

1-Cyanobiphenylene (**1-CNBP**) and 2-cyanobiphenylene (**2-CNBP**) were synthetized as shown below:

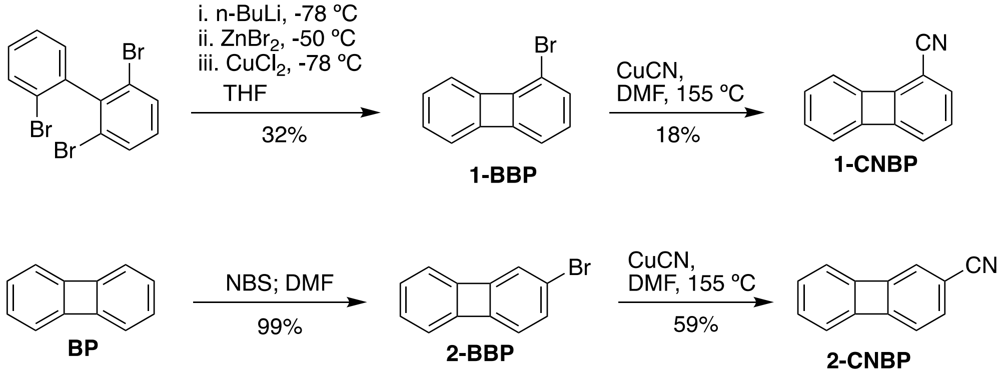

1.1.1. Synthesis of 1-bromobiphenylene (1-BBP).

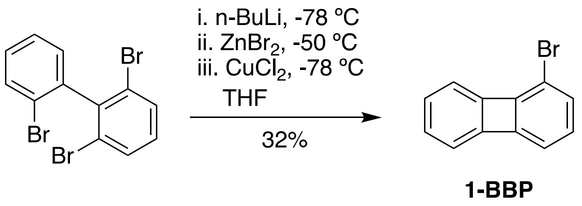

To a stirred solution of 2,2′,6-tribromobiphenyl[1] (750 mg, 1.91 mmol) in dry THF (25.6 mL), 2.2 M *n*-BuLi in hexane (1.8 mL, 4.02 mmol) was added at -78 ºC. The mixture was stirred for 2h at -78 ºC an then, a solution of $ZnBr_2$ (475.3 mg, 2.11 mmol) in THF (7.7 mL) was added and stirred for 2h at -50 ºC. Then, a solution of $CuCl_2$ (77.39 mg, 5.75 mmol) in THF (26.2 mL) was added over the curse of 3 h. Once the addition was completed, the mixture was stirred for 2 additional hours and then allowed to reach room temperature overnight. Then, the reaction was quenched with 10% aq. HCl (25 mL) and extracted with toluene (3 x 20 mL). The combined organic phases were dried with Na_2SO_4, filtered and evaporated under reduce pressure. The final residue was purified by column chromatography on silica gel (hexane) yielding 1-bromobiphenylene (**1-BBP**) as a yellow oil (166 mg, 32%). Analytical data were in accordance with literature. **^{1}H-NMR** (300 MHz, $CDCl_3$), δ: 6.79 (m, 1H), 6.69 – 6.55(m, 1H) ppm. **^{13}C-NMR-DEPT** (75 MHz, $CDCl_3$), δ: 152.78(C), 150.90(C), 149.74(C), 149.63(C), 131.40(CH), 129.98(CH), 128.96(CH), 128.82(CH), 117.90(CH), 117.61(CH), 115.96(CH), 109.91(C) ppm. **HRMS (APCI-DIP-TOF)**, for $C_{12}H_7Br$ (([M$^+$]) Calcd: 229.9726; Found: 229.9720.

1.1.2. Synthesis of 1-cyanobiphenylene (1-CNBP).

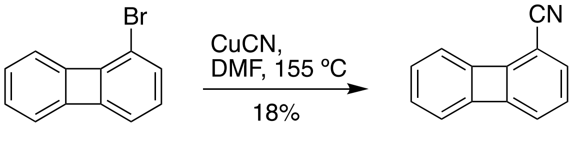

1-BBP **1-CNBP**

To a solution of 1-bromobiphenylene (**1-BBP**, 155 mg, 0.671 mmol) in dry DMF (8.9 mL), cuprous cyanide (180 mg, 2.01 mmol) was added and the mixture was heated at 155 ºC during 16h. Then, aqueous ammonia was added and the product was extracted with CH_2Cl_2 (3x15 mL). The combined organic layers were dried with Na_2SO_4, filtered and evaporated under reduced pressure. The crude residue was purified by column chromatography on silica gel (Hexane/ EtOAc, 9:1) yielding compound **1-CNBP** as a pale yellow solid (18 mg, 18%). **^{1}H-NMR** (300 MHz, $CDCl_3$), δ: 6.88 (m, 4H), 6.83-6.69 (m, 3H) ppm. **^{13}C-NMR-DEPT** (75 MHz, $CDCl_3$) δ: 156.40(C), 151.71(C), 150.24(C), 148.18(C), 130.46(CH), 129.54(CH), 129.01(CH), 128.66(CH), 119.73(CH), 119.34(CH), 118.58(CH), 116.15(C), 101.21(C) ppm. **HRMS (APCI-DIP-TOF)** for $C_{13}H_7N$ ([M^+]) Calcd.: 177.0576; Found: 177.0573

1.1.3. Synthesis of 2-bromobiphenylene (2-BBP).

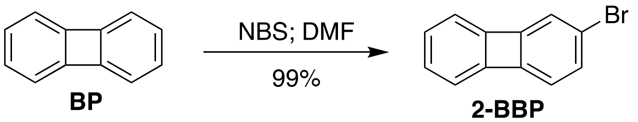

BP **2-BBP**

To a stirred solution of biphenylene (**BP**, 200 mg, 1.31 mmol) in dry DMF (3.3 mL), neat NBS (257.3 mg, 1.44 mmol) was added. The solution was stirred overnight a room temperature and then transferred to a flask with ice-cold water. The precipitate was filtered and washed with a large amount of water to remove the residual DMF. The compound was taken up with EtOAc, concentrated under reduce pressure and filtered through a short plug of silica gel (hexane) to afford 2-bromobiphenylene (**2-BBP**) as a yellow oil (300 mg, 99%). Analytical data were in accordance with literature.[1] **^{1}H-NMR** (300 MHz, $CDCl_3$), δ: 6.90 (dd, J = 7.3, 1.5 Hz, 1H), 6.80 – 6.74 (m, 3H), 6.65 (m, 2H), 6.51 – 6.46 (m, 1H) ppm.**^{13}C-NMR-DEPT** (75 MHz, $CDCl_3$), δ: 152.87(C), 150.08(C), 149.94(C), 130.66(CH), 129.14(CH), 128.71(CH), 121.65(C), 121.29(CH), 118.77(CH), 118.21(CH), 118.01(CH) ppm. **HRMS (APCI-DIP-TOF)** for $C_{12}H_7Br$ ([M^+]) Calcd.: 229.9726; Found: 229.9719.

1.1.4. Synthesis of 2-cyanobiphenylene (2-CNBP).

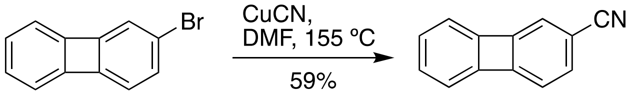

To a solution of 2-bromobiphenylene (**2-BBP**, 280 mg, 1.21 mmol) in dry DMF (12.1 mL), cuprous cyanide (434 mg, 4.84 mmol) was added and the mixture was heated at 155 °C during 16h. Then, aqueous ammonia was added and the product was extracted with CH_2Cl_2 (3 x 15 mL). The combined organic layers were dried with Na_2SO_4, filtered and evaporated under reduced pressure. The crude residue was purified by column chromatography on silica gel (Hexane/ EtOAc, 9:1) yielding 2-cyanobophenylene (**2-CNBP**) as a pale yellow solid (100 mg, 59%). **^{1}H-NMR** (300 MHz, $CDCl_3$), δ: 7.14 (dd, J = 7.1, 1.2 Hz, 1H), 6.92 – 6.87 (m, 2H), 6.81 – 6.75 (m, 3H), 6.70 (d, J = 1.0 Hz, 1H) ppm. **^{13}C-NMR-DEPT** (75 MHz, $CDCl_3$), δ: 156.08(C) 152.04(C), 149.70(C), 149.57(C), 135.07(CH), 130.09(CH), 129.57(CH), 119.22(CH), 118.88(CH), 118.24(CH), 117.06(CH), 111.28(C), 29.69(C) ppm. **HRMS (APCI-DIP-TOF)** for $C_{13}H_1N$ ([M^+]) Calcd.: 177.0576; Found: 177.0573.

2. ^{1}H and ^{13}C NMR spectra

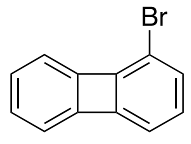

1-BBP

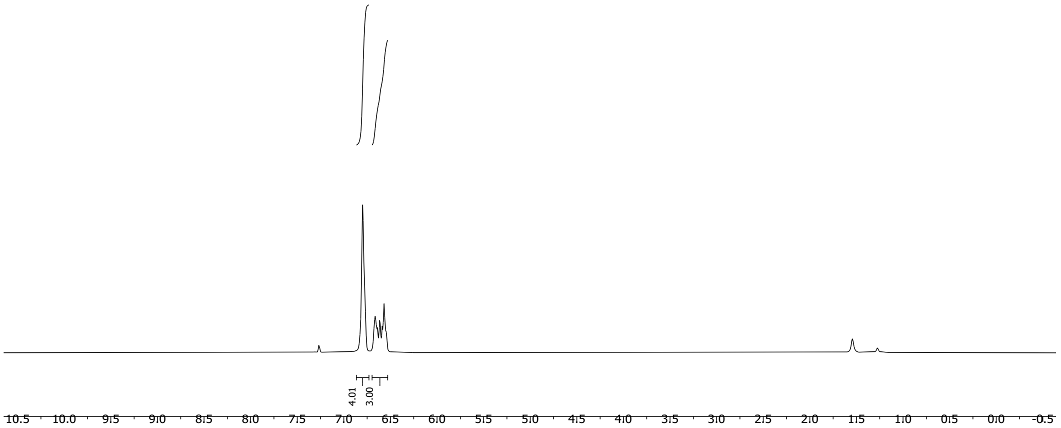

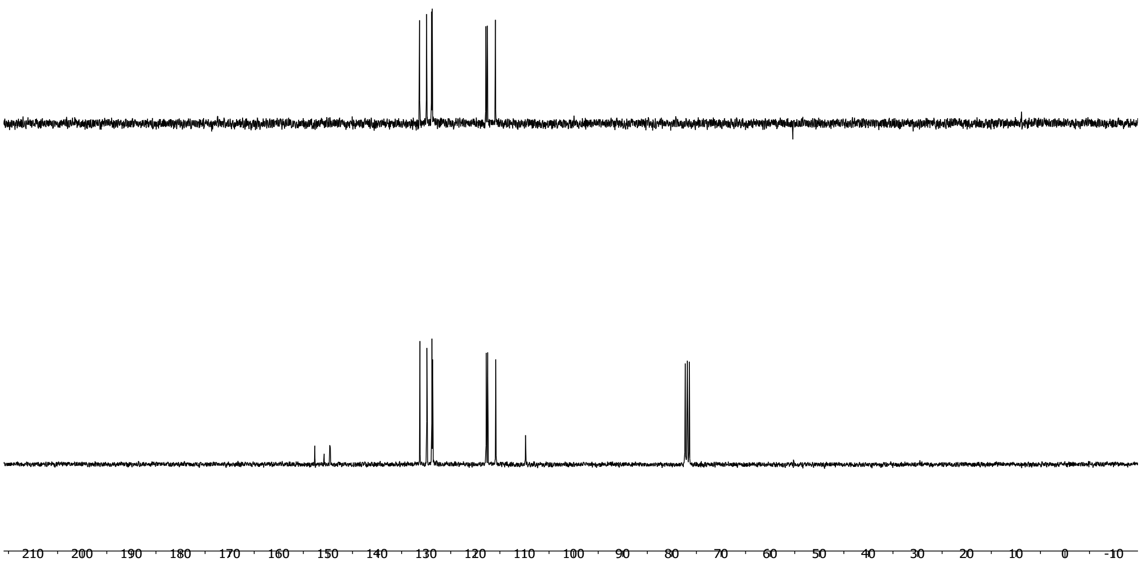

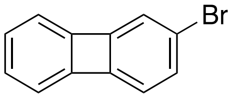

2-BBP

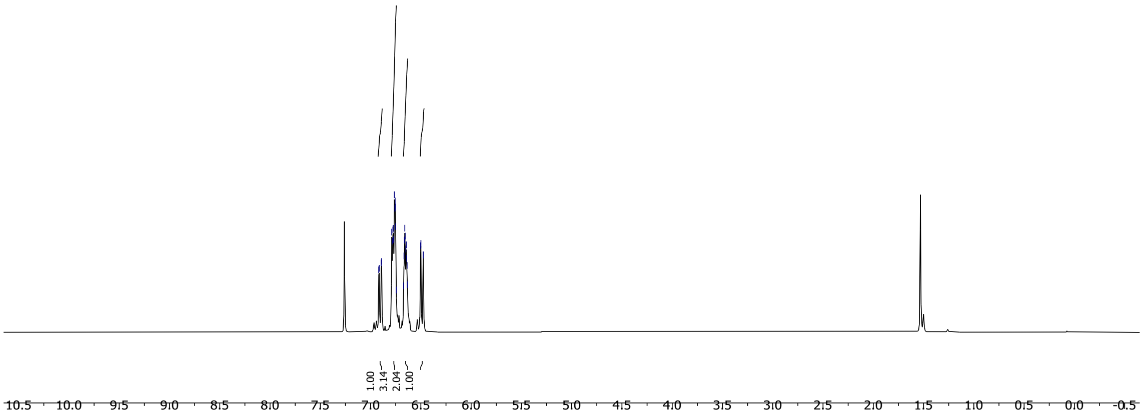
1.00
3.14
2.04
1.00
10.5 10.0 9.5 9.0 8.5 8.0 7.5 7.0 6.5 6.0 5.5 5.0 4.5 4.0 3.5 3.0 2.5 2.0 1.5 1.0 0.5 0.0 -0.5

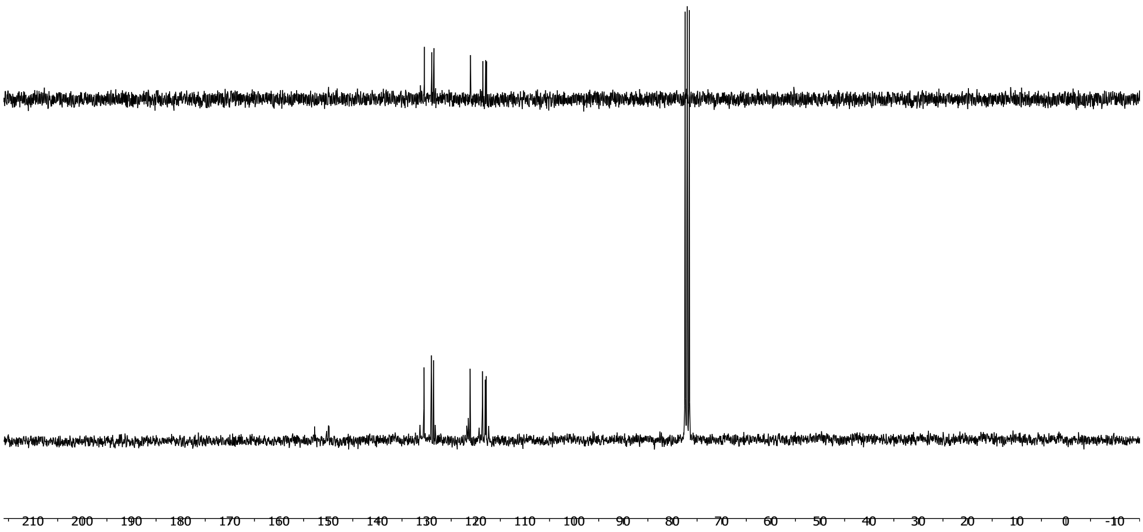
210 200 190 180 170 160 150 140 130 120 110 100 90 80 70 60 50 40 30 20 10 0 -10

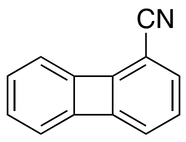
CN

1-CNBP

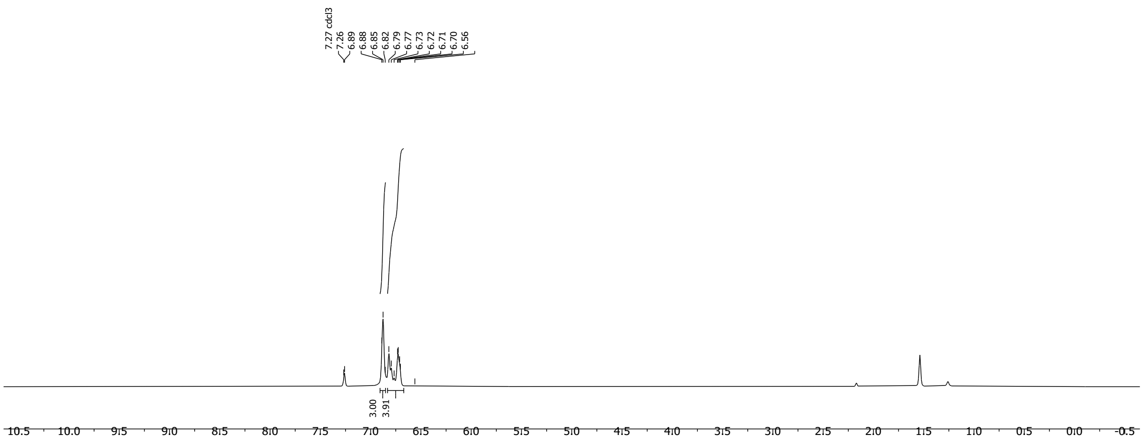
7.27 cdcl3
7.26
6.89
6.88
6.85
6.82
6.79
6.77
6.73
6.72
6.71
6.70
6.56
3.00
3.91
10.5 10.0 9.5 9.0 8.5 8.0 7.5 7.0 6.5 6.0 5.5 5.0 4.5 4.0 3.5 3.0 2.5 2.0 1.5 1.0 0.5 0.0 -0.5

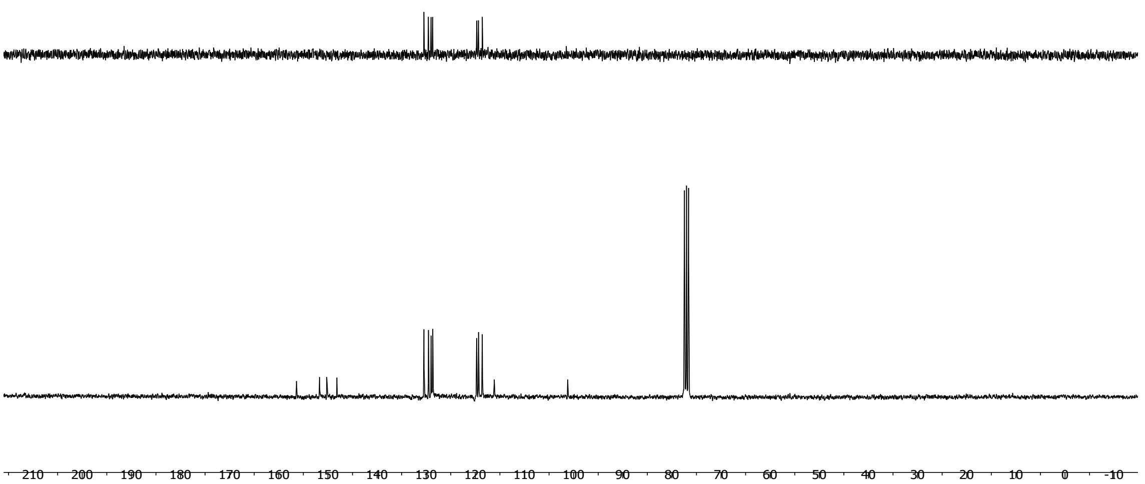
210 200 190 180 170 160 150 140 130 120 110 100 90 80 70 60 50 40 30 20 10 0 -10

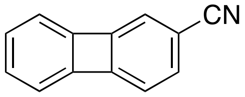

2-CNBP

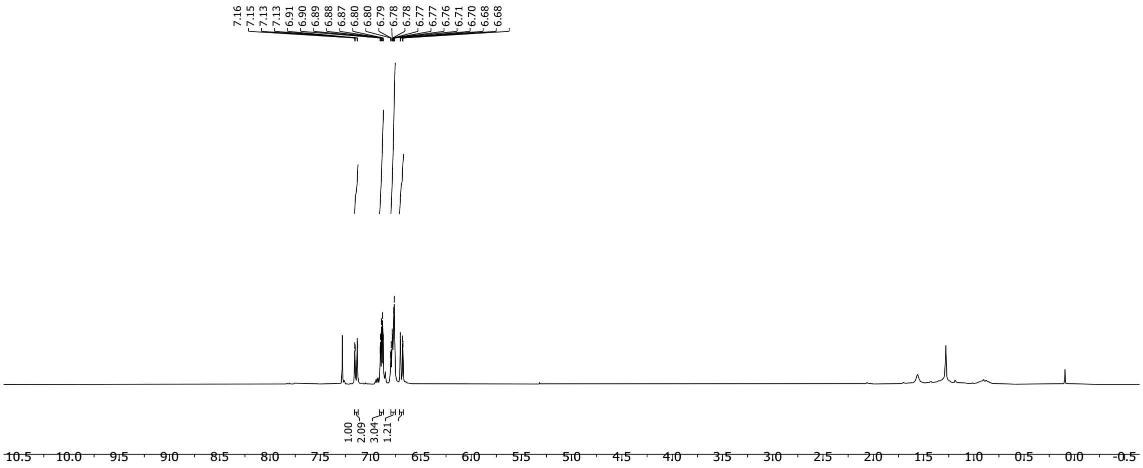

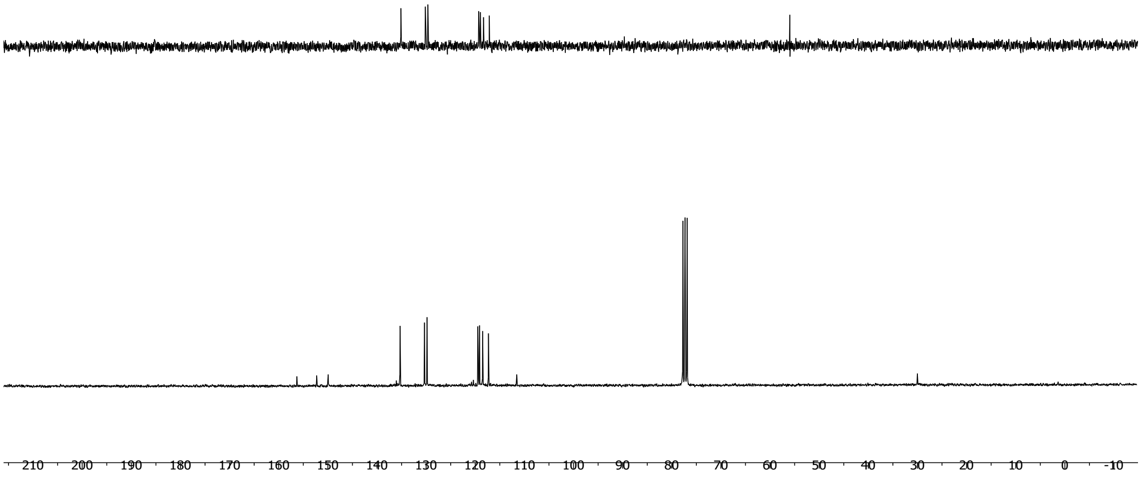

3. Selected UV-Vis and fluorescence spectra.

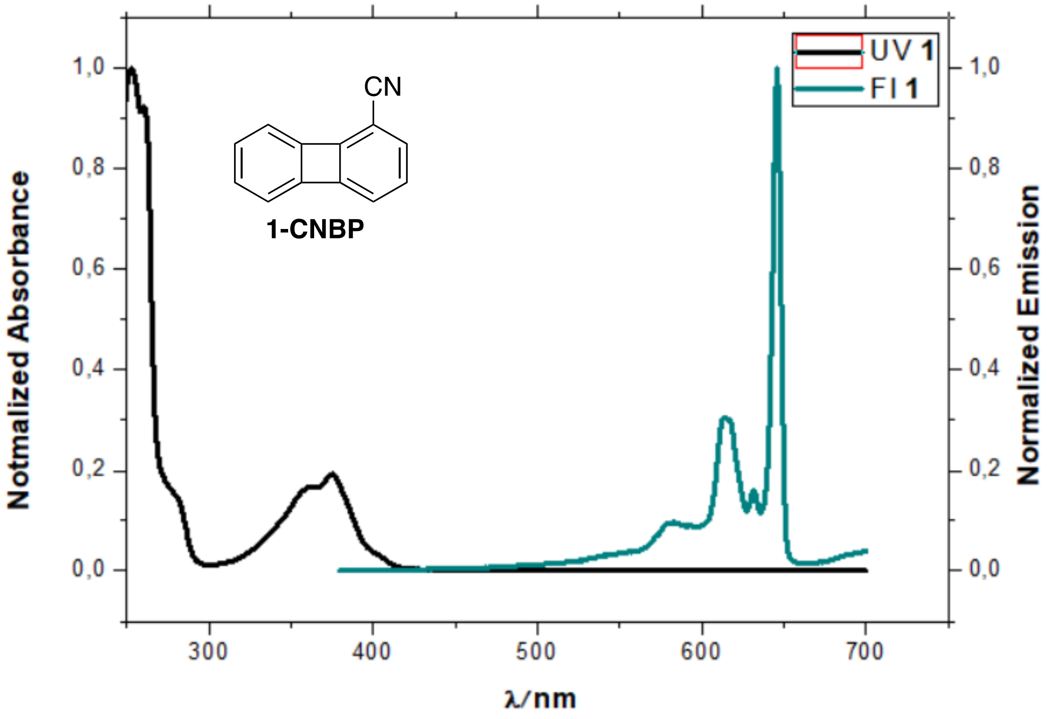

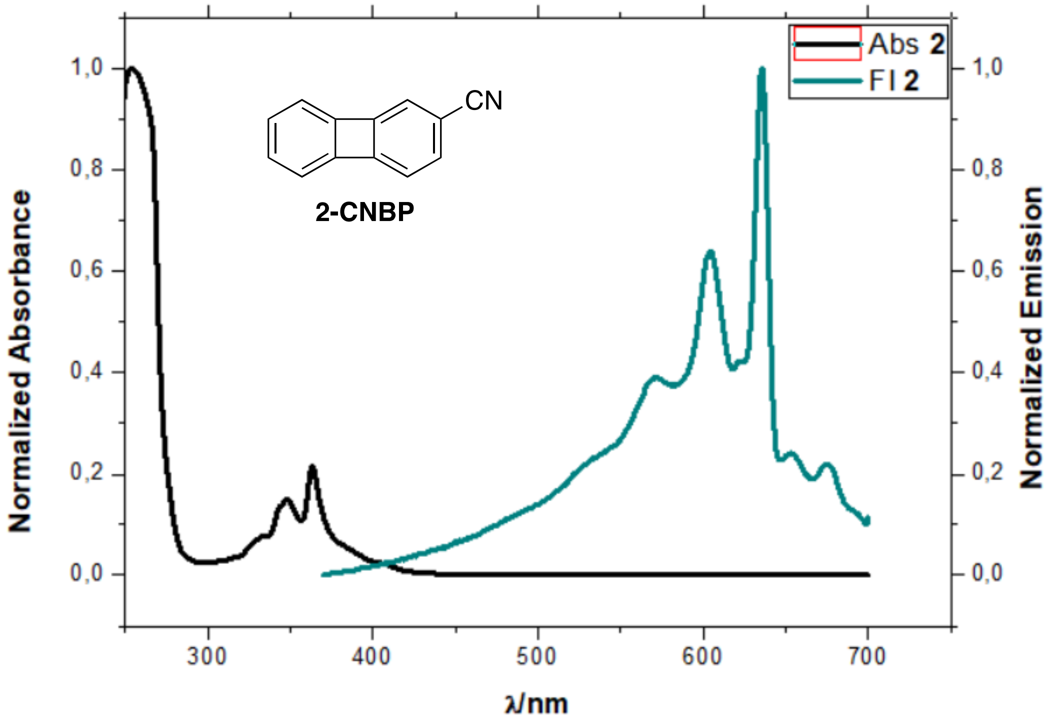

Structural Methods

The determination of the equilibrium structures (r_e) of 1-cyano and 2-cyanobiphenylene was conducted independently using structurally corrected *ab initio* calculations and the Watson's mass-dependent method[2] ($r_m^{(2)}$).

1. **Structurally-corrected *ab initio* estimation of the equilibrium structure**

The first estimation of the equilibrium structure used second-order Moller-Plesset[3] perturbation theory (MP2) and a correlation-consistent triple-ζ basis set from Dunning (cc-pVTZ[4]). These calculations are shown in Tables S1 and S2. The MP2/cc-pVTZ level of theory is able to predict the bond angles with an accuracy better than 0.3-0.4° in most cases.[5] The *ab initio* structures then serve as starting point for the calculation of predicates which will later be used in the mass-averaged structure of section 2 below.

For the carbon-hydrogen (CH) bond lengths, the MP2/cc-pVTZ method typically delivers values with accuracies better than 0.001 Å (see also Tables S1-S2).[6,7]

The case of the carbon-carbon (CC) bond is more complicated because the range of its length is large in the title compounds. Furthermore, the differences r_e(CC) - r(MP2/cc-pVTZ) are not constant because of the differences in hybridization of the carbon orbitals participating in the molecular bonds.[6,7] However, a plot of this difference as a function of r_e shows that the difference varies almost linearly.[6,7] From the *ab initio* calculations, it appears that the CC bond lengths in cyanobiphenylene are intermediate between double bonds and single bonds. For this category of partial double bonds, the equilibrium value is often longer than the MP2/cc-pVTZ value (see Table S3). A linear regression of the values of 40 molecules in Figure S2 gives the following result:

$$r_e(\mathrm{C{-}C}) = 0.9653(66) \times r[\mathrm{MP2/cc\text{-}pVTZ}] + 0.0533(96) \quad \text{(S1)}$$

with a correlation coefficient of 0.9982 and a standard deviation of the fit of 0.0017 Å. The sample of reference data is given in Table S3. There is however, an important exception: the CC bond lengths in a phenyl ring where the equilibrium value is shorter than the MP2/cc-pVTZ value.[8] In this case, a linear regression of sixteen equilibrium distances (Table 1 of reference 8) gives:

$$r_e(\mathrm{C \overset{...}{-} C}) = 0.998414(73) \times r[\mathrm{MP2/cc\text{-}pVTZ}] \quad \text{(S2)}$$

with a correlation coefficient of 0.9999999 and a standard deviation of the fit of 0.00055 Å. This correlation has been found valid for bond lengths in aromatic molecules, but as

cyanobiphenylene is antiaromatic, it is not obvious that it can be used. There are at least two ways to make sure that it can be applied in our case:

i) A comparison of results with the r_s values when they are believed to be accurate, see Tables S1-S2 (final structures). In particular, for the bond length C4b-C5, equation (S2) gives 1.372 Å whereas the r_s value is 1.372(1) Å.

ii) A more stringent confirmation will be given by the r_m-fit of the next section which may be used to examine the compatibility of the predicates (i.e. the corrected MP2/cc-pVTZ values) with the rotational constants. For the CC ring bonds, it will be shown that, when there is a large discrepancy, the fitted value is smaller than the predicate value.

This procedure confirms that it is better to use Eq. (S2) for the CC ring bonds.

For the side-chain carbon-carbon (C1Cα or C2Cα) bonds, Eq. (S1) may be used. It gives 1.431(2) Å for 1-cyano and 1.433(2) for 2-cyanobiphenylene, respectively.

Concerning the carbon-carbon bridge (C4a-C4b) bond length, the situation is easier because, although it is between two multiple bonds, it behaves as a single bond. An Atoms-in-Molecules (AIM) calculation for 1-cyanobiphenylene indicates that the ellipticity[9] for this bond is only 0.037 whereas it is 0.215 for C2C3 and 0.150 for C4aC8b, see Tables S4-S5. For 2-cyanobiphenylene, the ellipticity is 0.038. As shown in Table S1, for such a long bond, the MP2/cc-pVTZ value of 1.5072 Å for 1-cyanobiphenylene is close to the equilibrium values. Likewise, for 2-cyanobiphenylene in Table S2, the MP2/VTZ value is 1.503 Å to be compared with the equilibrium value of 1.502(2) Å.

Finally, for the carbon-nitrogen (CN) triple-bond length the situation is still easier because the variation range of the equilibrium distances is small. Therefore, it may be assumed that the correction is constant. A selection of equilibrium values for CN bonds is shown in Table S6.

The accuracy of the structural calculations can be checked qualitatively by comparison with the substitution structure[10] method (r_s), provided that a selection of atoms is chosen with large cartesian coordinates. The substitution structures are included in Tables S1-S2. The uncertainties in the substitution structure calculations have been discussed elsewhere.[11] A satisfactory comparison of some bond length predicates with the substitution structure is shown in Table S7.

2. Mass-dependent method

The mass-dependent structural method of Watson *et al.*[2] (r_m) permits obtaining a very accurate equilibrium structure for small molecules from the zero-point moments of inertia of a set of

isotopologues. Unfortunately, the Watson method has considerable requirements and often fails for large molecules. This is mainly due to the fact that the number of structural parameters to fit can be very large, requiring a considerable number of isotopic species. Therefore, the system of normal equations of the least-squares fit may be ill-conditioned. However, an easy way to remedy this difficulty is to use the method of mixed regression,[12] where the rotational constants are supplemented by structural parameters from *ab initio* calculations, here from the initial MP2/cc-pVTZ structurally corrected predictions of section 1.

The r_m method was already employed with success for several moderately large molecules like ethynylcyclohexane (C_8H_6),[13] diallyl disulfide ($C_6H_{10}S_2$),[14] diphenyl disulfide ($C_{12}H_{10}S_2$),[8] or fructose ($C_6H_{12}O_6$).[15] The determination of the Watson r_m structure for the cyanobiphenylenes ($C_{13}H_7N$) represents a considerable advance because of the structural interest of the molecule, probably one of the largest for which this method was attempted.

The Watson method relates the ground-state and equilibrium moments of inertia by either explicit functions of degree ½ on the equilibrium moments of inertia ($r_m^{(1)}$ method) or by a two-parameter model including an additional 1/(2*N*-2) power dependence on the reduced atomic masses ($r_m^{(2)}$ method). In this way, the ground-state moment of inertia I_0^ξ for each inertial axis ξ can be approximated by

$$I_0^\xi = I_{\mathrm{m}}^\xi + c_\xi \sqrt{I_{\mathrm{m}}^\xi} + d_\xi \left(\frac{m_1 m_2 \cdots m_n}{M}\right)^{1/(2N-2)} \quad \text{(S3)}$$

In this equation, *N* is the number of atoms, m_i their respective masses and c_ξ and d_ξ are the two fitting rovibrational parameters.

The $r_m^{(2)}$ method has two advantages:

i) It allows to obtain a reliable structure without too much computational work, as there is no need to calculate rovibrational corrections for all isotopologues (all the more so that it does not solve the problem of ill-conditioning)

ii) It permits to check that the experimental rotational constants are compatible with the (corrected) *ab initio* structure.

The statistical diagnostics for the $r_m^{(2)}$ fit use the Studentized residual to detect outliers and the diagonal elements of the H_{at} matrix which permit to detect leverage values (a leverage is high when a small change of the input value causes a large change in the solution).[16] In the present case we used fifteen isotopologues for each isomer (45 moments of inertia), and fitted the molecular structure assuming only the planarity of the ring system and the nitrile group.

The fit results indicate that there is no outlier and that the weighting was adequate. However, the condition number is high, $\kappa = 1.1\times10^5$. Therefore, some parameters may be less

accurate than indicated by their standard deviation.[12] The analysis of the variance-decomposition proportions shows that this problem does not affect the bond lengths but only a few bond angles. This is fortunate because, as shown below, it is relatively easy to confirm their accuracy. However, some rovibrational parameters are poorly determined, see Table S1. For this reason, it was assumed that the fitting parameters for the *b* and *c* axes are the same ($c_b = c_c$ and $d_b = d_c$). As shown in Table S1, these constraints do not affect the values of the parameters.

In the final fit, the internal coordinates of the hydrogen atoms where kept fixed at their MP2/cc-pVTZ values. This simplification may bias the parameters and affect their standard deviation. For this reason, the fit was repeated for 1-cyanobiphenylene freeing all the parameters and using the MP2/cc-pVTZ values as predicates for the coordinates of the hydrogen atoms. The new parameters from this global fit are almost identical to those of the reduced fit and their standard deviations are not increased, see Table 1.

3. Discussion

The quality of the final structures in Tables S1 and S2 is discussed below. A first check was made by calculating the rovibrational corrections for 2-cyanobiphenylene with the help of the *ab initio* MP2/cc-pVTZ anharmonic force field. The results (in MHz): $\Delta A = A_e - A_0 = 17.033$; $\Delta B = 1.708$; $\Delta C = 1.533$ are in reasonable agreement with the values deduced from the $r_m^{(2)}$ fit: $\Delta A = 17.644$; $\Delta B = 1.747$; $\Delta C = 1.568$.

3.1. Analysis of the bond angles

There are several ways to check the accuracy of the bond angles

3.1.1. Ab initio optimizations

The quality of the *ab initio* optimizations was discussed in section 1.

3.1.2. Effective (r_0) structure

Contrary to the r_0 bond lengths, the effective bond angles ($\angle_0$) are often a good approximation of the equilibrium values. An analysis of 45 angles gave a median absolute deviation (MAD) of 0.2° corresponding to a standard deviation of 0.3°.[17] For 1-cyanobiphenylene, the MAD is 0.25° corresponding to a standard deviation of 0.36°, the largest deviation, 1 °, being for the C7C8C8a bond angle. However, the r_0 value, 116.1(18)°, is rather inaccurate. Furthermore, for this angle there is a good agreement between the $r_m^{(2)}$ value and the MP2/cc-pVTZ value. Finally, the effective and $r_m^{(2)}$ values of the ∠(C4C4aC8b) angle are also in good agreement. This a further indication that the $r_m^{(2)}$ angles are accurate. For 2-

cyanobiphenylene, the MAD is 0.4° corresponding to a standard deviation of 0.6°, the largest deviation being 0.7°, for the ∠(C4a-C8b-C1) angle, but this is smaller than the standard deviation of the $\angle_0$ value, 0.8°.

3.1.3. Zero-point average structure (r_α)

The $\angle_\alpha$ (or $\angle_z$) angles are also a good approximation of the equilibrium values, the difference being generally smaller than 0.2°.[17] Although the $\angle_\alpha$ angles of biphenylene are not precise, they are in good agreement with our results. In particular, the $\angle_\alpha$(C4aC4bC5) value of biphenylene, 147.5(6)° is in excellent agreement with the $r_m^{(2)}$ value of 147.52(5)° of 1-cyanobiphenylene.

3.2. Analysis of the bond lengths

The situation for the bond lengths is more complicated. First, the comparison with the r_s structure does not help because some Cartesian coordinates of the atoms C4a and C8b are quite small (a[C4a] = 0.066 Å and b[C8b] = 0.041 Å). For this reason, the r_s value of the bond length C4aC8b is inaccurate.

For the CC bonds, it is possible to calculate the electronic density at the bond critical point with the Atom in Molecules method.[9] (see Figure S3 and Tables S4 and S5). This parameter, ρ_b, gives the amount of electron density shared between the two bonded atoms and is roughly proportional to the bond length. Indeed, a plot of the $r_m^{(2)}$ CC bond lengths as a function of ρ_b in Figure S4 shows a good correlation (correlation coefficient, 0.995) for 1-cynobiphenylene and, thus, confirms the reliability of the $r_m^{(2)}$ structure. Figure S4 clearly shows three almost linear sections: first, three points corresponding to long CC bonds C8aC8b, C4aC4b, and C1Cα. For these bonds, $\varepsilon \leq 0.7$. Then, bonds with $0.15 \leq \varepsilon \leq 0.16$ whose length is between 1.411 Å and 1.417 Å. Finally, the third section $0.20 \leq \varepsilon \leq 0.22$ whose length is between 1.371 Å and 1.393 Å. Another indication of the reliability of the $r_m^{(2)}$ structure is that, as discussed above, all the bond angles are accurate. Figure S5 shows a similar representation for 2-cyanobiphenylene.

Upper limit of column density calculations

To compute upper limits to the column densities of 1-CNBP and 2-CNBP in TMC-1, we proceeded in this way. We first predict the line intensities under local thermodynamic equilibrium (LTE) conditions for the rotational temperature adopted and identify the lines that are predicted to be the most intense ones in the Q band. These correspond to the three lines shown in Figure 3. We then compute the 3σ upper limits to their velocity-integrated line intensity as:

$$\int T_A^* dv\ (3\sigma) = 3 \times rms \times \sqrt{\frac{\delta v}{\Delta v}}\ \Delta v,$$

where rms is the noise level measured in the observed spectrum in the spectral region around each line (in antenna temperature scale and units of mK), δv is the spectral resolution of the spectrum in velocity (with units of km s^{-1}), and Δv is the full width at half maximum expected for the line (here taken as 0.60 km s^{-1} for TMC-1). We then calculate the column density that would be needed to account for the 3σ velocity-integrated intensity derived for each line in the previous stage, and adopt as 3σ upper limit to the column density the smallest value. In the case of 1-CNBP, the most stringent upper limit is provided by the $14_{14,1}$-$13_{13,0}$ + $14_{,14,0}$-$13_{13,1}$ lines at 34025.132 MHz, while for 2-CNBP the upper limit to its column density is set by the $43_{4,39}$-$42_{4,38}$ line at 31851.719 MHz.

REFERENCES

(1) Kirschner, S.; Uecker, I.; Bolte, M.; Lerner, H.-W.; Wagner, M. How π Extension or Structural Bending Alters the Properties of Boron-Doped Phenylene-Containing Oligoacenes. *Organometallics* **2019**, *38* (14), 2818–2823. https://doi.org/10.1021/acs.organomet.9b00330.

(2) Watson, J. K. G.; Roytburg, A.; Ulrich, W. Least-Squares Mass-Dependence Molecular Structures. *J Mol Spectrosc* **1999**, *196* (1), 102–119. https://doi.org/10.1006/jmsp.1999.7843.

(3) Møller, C.; Plesset, M. S. Note on an Approximation Treatment for Many-Electron Systems. *Physical Review* **1934**, *46* (7), 618–622. https://doi.org/10.1103/PhysRev.46.618.

(4) Peterson, K. A.; Dunning, T. H. Accurate Correlation Consistent Basis Sets for Molecular Core–Valence Correlation Effects: The Second Row Atoms Al–Ar, and the First Row Atoms B–Ne Revisited. *J Chem Phys* **2002**, *117* (23), 10548–10560. https://doi.org/10.1063/1.1520138.

(5) Margulès, L.; Demaison, J.; Boggs, J. E. Ab Initio and Equilibrium Bond Angles. Structures of HNO and H2O2. *Journal of Molecular Structure: THEOCHEM* **2000**, *500* (1–3), 245–258. https://doi.org/10.1016/S0166-1280(00)00371-7.

(6) Demaison, J. F.; Craig, N. C. Semiexperimental Equilibrium Structure for Cis , Trans - 1,4-Difluorobutadiene by the Mixed Estimation Method. *J Phys Chem A* **2011**, *115* (27), 8049–8054. https://doi.org/10.1021/jp203575r.

(7) Demaison, J.; Craig, N. C.; Cocinero, E. J.; Grabow, J.-U.; Lesarri, A.; Rudolph, H. D. Semiexperimental Equilibrium Structures for the Equatorial Conformers of N - Methylpiperidone and Tropinone by the Mixed Estimation Method. *J Phys Chem A* **2012**, *116* (34), 8684–8692. https://doi.org/10.1021/jp304178n.

(8) Demaison, J.; Vogt, N.; Saragi, R. T.; Juanes, M.; Rudolph, H. D.; Lesarri, A. The S−S Bridge: A Mixed Experimental-Computational Estimation of the Equilibrium Structure of Diphenyl Disulfide. *ChemPhysChem* **2019**, *20* (3), 366–373. https://doi.org/10.1002/cphc.201800973.

(9) Gillespie, R. J.; Popelier, P. L. A. *Chemical Bonding and Molecular Geometry*; Oxford University Press: Oxford, 2001.

(10) Kraitchman, J. Determination of Molecular Structure from Microwave Spectroscopic Data. *Am J Phys* **1953**, *21* (1), 17–24. https://doi.org/10.1119/1.1933338.

(11) Demaison, J.; Rudolph, H. D. When Is the Substitution Structure Not Reliable? *J Mol Spectrosc* **2002**, *215* (1), 78–84. https://doi.org/10.1006/jmsp.2002.8610.

(12) Belsey, A. *Conditioning Diagnostics: Collinearity and Weak Data in Regression*; Wiley: New York, NY, 1991.

(13) Vogt, N.; Demaison, J.; Rudolph, H. D.; Juanes, M.; Fernández, J.; Lesarri, A. Semiexperimental and Mass-Dependent Structures by the Mixed Regression Method: Accurate Equilibrium Structure and Failure of the Kraitchman Method for Ethynylcyclohexane. *Journal of Chemical Physics* **2018**, *148* (6), 1–11. https://doi.org/10.1063/1.5018053.

(14) Demaison, J.; Vogt, N.; Saragi, R. T.; Juanes, M.; Rudolph, H. D.; Lesarri, A. How Flexible Is the Disulfide Linker? A Combined Rotational-Computational Investigation of Diallyl Disulfide. *Physical Chemistry Chemical Physics* **2019**, *21* (36), 19732–19736. https://doi.org/10.1039/c9cp02508a.

(15) Vogt, N.; Demaison, J.; Cocinero, E. J.; Écija, P.; Lesarri, A.; Rudolph, H. D.; Vogt, J. The Equilibrium Molecular Structures of 2-Deoxyribose and Fructose by the Semiexperimental Mixed Estimation Method and Coupled-Cluster Computations.

Physical Chemistry Chemical Physics **2016**, *18* (23), 15555–15563. https://doi.org/10.1039/c6cp01842d.
(16) Demaison, J. The Method of Least-Squares. In *Equilibrium Molecular Structures*; Demaison, J., Boggs, J. E., Csaszar, A. G., Eds.; CRC Press: Boca Raton, Florida, 2011; pp 29–52. https://doi.org/10.1201/b10374.
(17) Rudolph, H. D.; Demaison, J. Determination of the Structural Parameters from the Inertial Moments. In *Equlibrium Molecular Structures*; Demaison, Jean; Boggs, J. ; Csaszar, A., Ed.; CRC Press: Boca Raton, Fl, 2011; pp 125–158.
(18) Botschwina, P.; Horn, M.; Seeger, S.; Flügge, J. A Theoretical Investigation of HC2NC and HNC3. *Chem Phys Lett* **1992**, *195* (4), 427–434. https://doi.org/10.1016/0009-2614(92)85629-O.
(19) Botschwina, P.; Flügge, J. Ab Initio Vibration—Rotation Coupling Constants and the Equilibrium Geometries of NCCN and CNCN. *Chem Phys Lett* **1991**, *180* (6), 589–593. https://doi.org/10.1016/0009-2614(91)85015-O.
(20) Thorwirth, S.; Harding, M. E.; Dudek, J. B.; McCarthy, M. C. Equilibrium Molecular Structures of Vinyl Carbon Chains: Vinyl Acetylene, Vinyl Diacetylene, and Vinyl Cyanide. *J Mol Spectrosc* **2018**, *350*, 10–17. https://doi.org/10.1016/j.jms.2018.05.001.
(21) Rudolph, H. D.; Demaison, J.; Császár, A. G. Accurate Determination of the Deformation of the Benzene Ring upon Substitution: Equilibrium Structures of Benzonitrile and Phenylacetylene. *J Phys Chem A* **2013**, *117* (48), 12969–12982. https://doi.org/10.1021/jp408208s.
(22) Puzzarini, C.; Cazzoli, G. Equilibrium Structure of Methylcyanide. *J Mol Spectrosc* **2006**, *240* (2), 260–264. https://doi.org/10.1016/j.jms.2006.10.005.
(23) Demaison, J.; Margulès, L.; Mäder, H.; Sheng, M.; Rudolph, H. D. Torsional Barrier and Equilibrium Structure of Ethyl Cyanide. *J Mol Spectrosc* **2008**, *252* (2), 169–175. https://doi.org/10.1016/j.jms.2008.08.002.
(24) Petitprez, D.; Wlodarczak, G.; Lignier, H.; Demaison, J.; de Meijere, A.; Steiniz, A. G.; Møllendal, H. The Microwave Stark and Fourier Transform Spectra, Structure and Quadrupole Coupling Constants of 1,2-Dicyanocyclobutene. *J Mol Struct* **2002**, *612* (2–3), 315–324. https://doi.org/10.1016/S0022-2860(02)00102-3.
(25) Barone, V.; Biczysko, M.; Bloino, J.; Cimino, P.; Penocchio, E.; Puzzarini, C. CC/DFT Route toward Accurate Structures and Spectroscopic Features for Observed and Elusive Conformers of Flexible Molecules: Pyruvic Acid as a Case Study. *J Chem Theory Comput* **2015**, *11* (9), 4342–4363. https://doi.org/10.1021/acs.jctc.5b00580.
(26) Müller, H. S. P.; Thorwirth, S.; Lewen, F. Rotational Spectroscopy of Singly 13C Substituted Isotopomers of Propyne and Determination of a Semi-Empirical Equilibrium Structure. *J Mol Struct* **2020**, *1207*, 127769. https://doi.org/10.1016/j.molstruc.2020.127769.
(27) Askeland, E.; Møllendal, H.; Uggerud, E.; Guillemin, J.-C.; Aviles Moreno, J.-R.; Demaison, J.; Huet, T. R. Microwave Spectrum, Structure, and Quantum Chemical Studies of a Compound of Potential Astrochemical and Astrobiological Interest: Z -3-Amino-2-Propenenitrile. *J Phys Chem A* **2006**, *110* (46), 12572–12584. https://doi.org/10.1021/jp064152d.
(28) Botschwina, P. Large-Scale Ab Initio Calculations of Spectroscopic Constants for CNCN. *Chem Phys Lett* **1994**, *225* (4–6), 480–485. https://doi.org/10.1016/0009-2614(94)87115-9.
(29) Carter, S.; Mills, I. M.; Handy, N. C. The Equilibrium Structure of HCN. *J Chem Phys* **1992**, *97* (2), 1606–1607. https://doi.org/10.1063/1.463237.
(30) Farkhsi, A.; Bredohl, H.; Dubois, I.; Remy, F.; Fayt, A. FT Infrared Spectra of FCN from 1200 to 1800 Cm−1 and from 2800 to 7000 Cm−1 and Global Rovibrational

Analysis of the Main Isotopomers. *J Mol Spectrosc* **2000**, *201* (1), 36–55. https://doi.org/10.1006/jmsp.2000.8084.
(31) Demaison, J.; Margulès., L.; Boggs, J. E. The Equilibrium C–Cl, C–Br, and C–I Bond Lengths from Ab Initio Calculations, Microwave and Infrared Spectroscopies, and Empirical Correlations. *Struct Chem* **2003**, *14*, 159–174. https://doi.org/10.1023/A:1022138431967.
(32) Demaison, J.; Herman, M.; Lievin, J. The Equilibrium OH Bond Length. *Int Rev Phys Chem* **2007**, *26* (3), 391–420. https://doi.org/10.1080/01442350701371919.

Figure S1. (a) Broadband microwave spectrum of 2-CNBP (2.8 million averages) in the region 2-8 GHz. (b) The nuclear quadrupole hyperfine structure of the $3_{2,2}$-$3_{1,3}$ rotational transition.

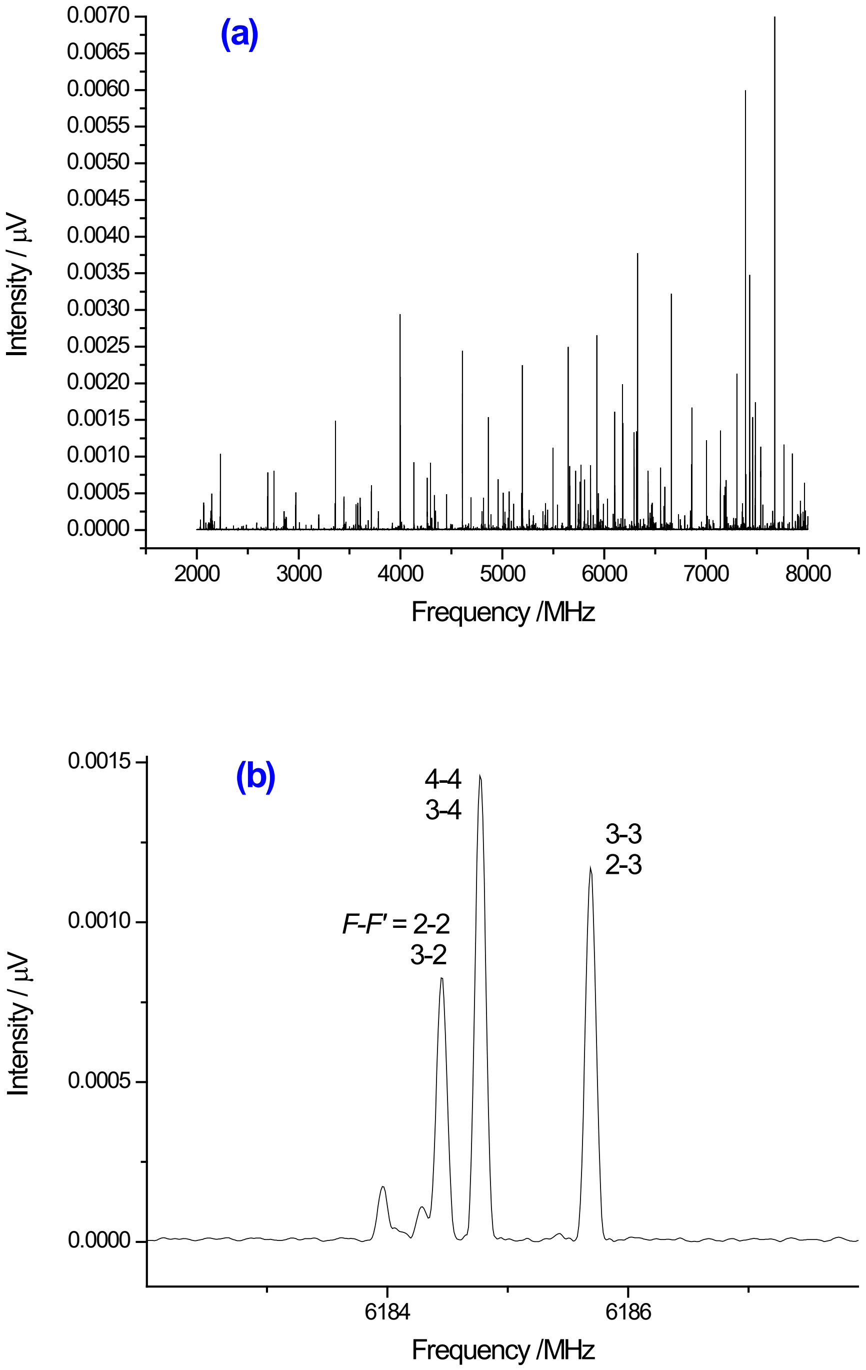

Figure S2. Correlation between the equilibrium carbon-carbon (C-C) bond distances r_e(CC) and the calculated values using MP2/cc-pVTZ (r_{MP2}).

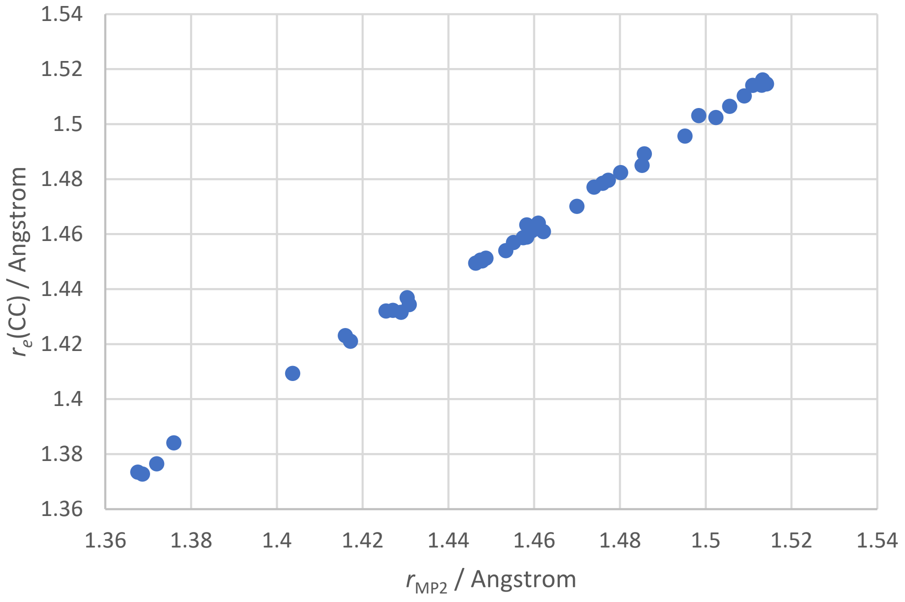

Figure S3. Atoms-in-Molecules (AIM) representation for 1-cyanobiphenylene showing critical points at the C-C and C-N bonds of the molecule. See Tables S4 and S5 for the electronic densities and ellipticities.

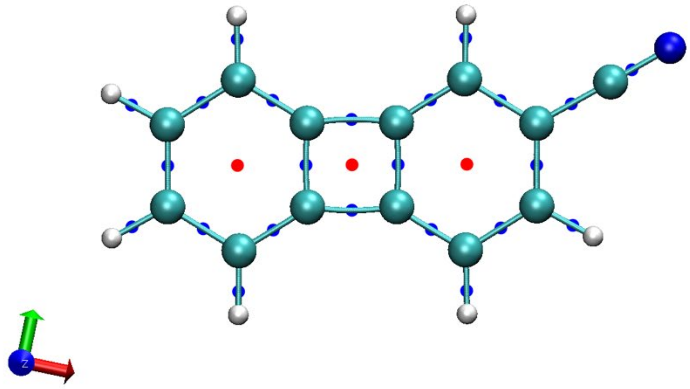

Figure S4. A representation of $r_m^{(2)}$ bond lengths (Å) vs. ρ_b (au) for 1-cyanobiphenylene.

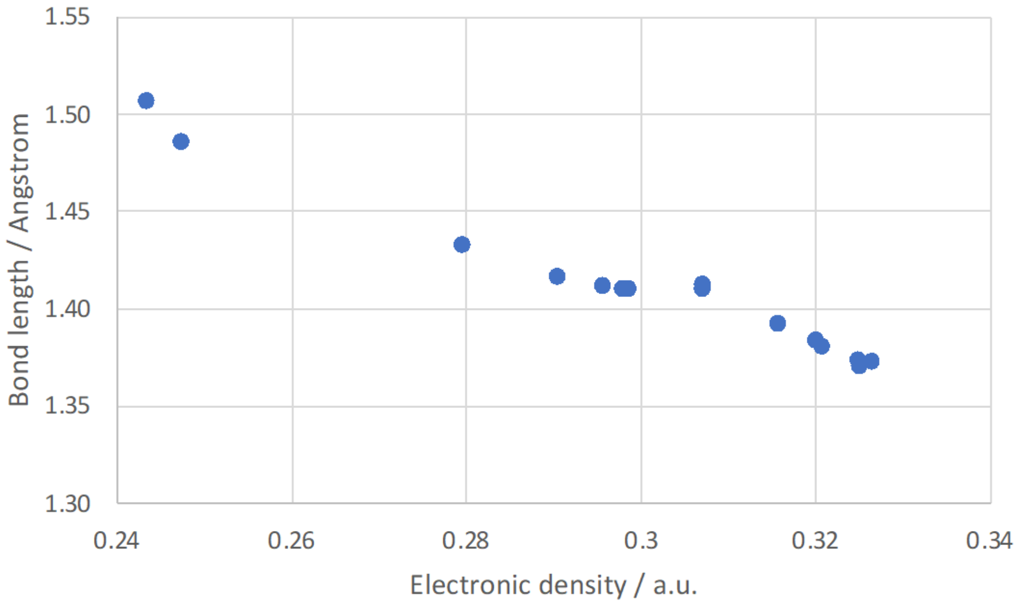

Figure S5. A representation of $r_{\mathrm{m}}^{(2)}$ bond lengths (Å) vs. ρ_b (au) for 2-cyanobiphenylene.

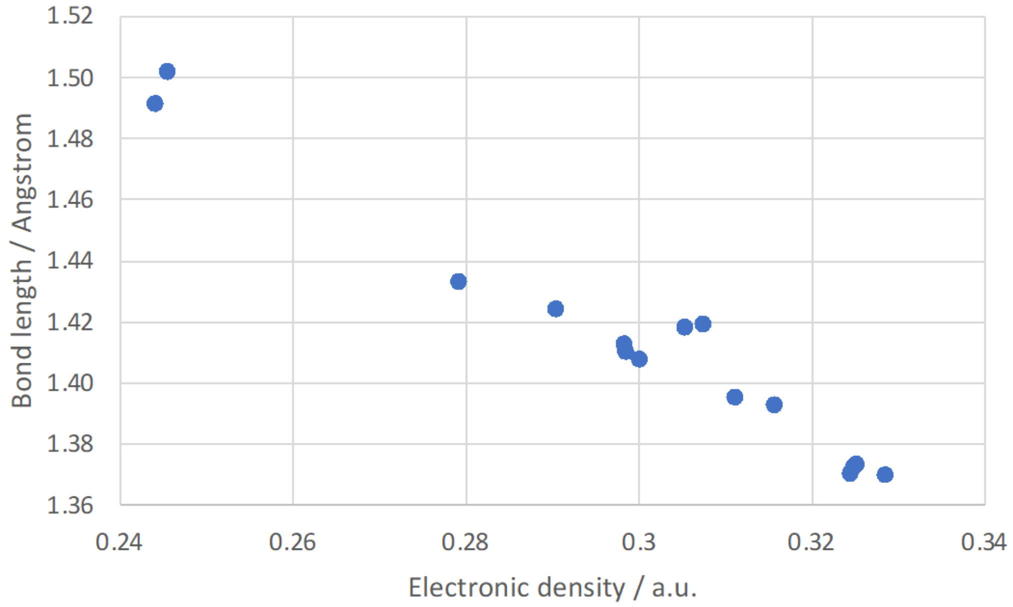

Table S1. Molecular structure of 1-cyanobiphenylene, including the *ab initio* calculations, the *ab initio* predicates, the mass-averaged equilibrium structure ($r_m^{(2)}$), the effective structure (r_0) and the substitution structure (r_s). The final equilibrium structure corresponds to the $r_m^{(2)}$ fit with four rovibrational parameters. In this fit all internal coordinates of the hydrogen atoms were fixed to the *ab initio* predicates. Alternative $r_m^{(2)}$ fits are shown for comparison, using six rovibrational parameters (Fit 2) or the internal hydrogen atom coordinates (Fit 3).

	MP2/ cc-pVTZ	Predicate	$r_m^{(2)}$ Final fit: 4 rovib [a]	$r_m^{(2)}$ Fit 2: 6 rovib. [b]	$r_m^{(2)}$ Fit 3: With H [a,c]	r_0	r_s
Bond lengths							
r(CN) / Å	1.1738	1.1588(20)[d]	1.15616(65)	1.15617(67)	1.15615(64)	1.158(13)	1.159(1)
r(CαC1) / Å	1.4269	1.4310(20)	1.4329(12)	1.4329(12)	1.4329(12)	1.430(20)	1.447(4)
r(C1C2) / Å	1.4192	1.4169(20)	1.4168(14)	1.4167(14)	1.4167(13)	1.433(20)	1.420(4)
r(C2C3) / Å	1.3890	1.3868(20)	1.3840(10)	1.3843(10)	1.3839(10)	1.384(21)	1.389(2)
r(C3C4) / Å	1.4140	1.4118(20)	1.4121(16)	1.4119(16)	1.4119(15)	1.419(21)	1.424(2)
r(C4C4a) / Å	1.3752	1.3730(20)	1.3730(13)	1.3726(14)	1.3729(14)	1.381(21)	1.390(26)
r(C4aC8b) / Å	1.4165	1.4143(80)	1.4131(16)	1.4132(16)	1.4130(16)	1.392(75)	1.450(21)
r(C4aC4b) / Å	1.5072	1.5070(20)	1.5070(16)	1.5066(16)	1.5070(16)	1.498(52)	1.483(39)
r(C4bC5) / Å	1.3746	1.3724(20)	1.3710(15)	1.3706(16)	1.3711(15)	1.381(21)	1.372(1)
r(C5C6) / Å	1.4123	1.4101(20)	1.4103(13)	1.4102(13)	1.4103(13)	1.419(21)	1.444(22)
r(C6C7) / Å	1.3937	1.3915(20)	1.3929(13)	1.3930(14)	1.3930(13)	1.384(21)	1.361(26)
r(C7C8) / Å	1.4116	1.4094(20)	1.4107(15)	1.4106(15)	1.4108(15)	1.433(20)	1.423(1)
r(C8C8a) / Å	1.3749	1.3727(20)	1.3738(16)	1.3737(16)	1.3740(16)	1.383(27)	1.381(3)
Bond angles							
∠(CαC1C2) / deg	121.53	121.53(30)	121.24(11)	121.29(12)	121.24(11)	121.3(8)	120.3(1)
∠(C1C2C3) / deg	121.26	121.26(30)	121.00(12)	121.01(12)	121.00(11)	121.2(9)	120.6(1)
∠(C2C3C4) / deg	122.52	122.52(30)	122.355(88)	122.363(89)	122.352(86)	122.6(6)	122.19(7)
∠(C3C4C4a) / deg	115.62	115.62(30)	115.943(70)	115.905(82)	115.936(72)	115.9(12)	117(1)
∠(C4C4aC8b) / deg	122.42	122.42(30)	122.61(21)	122.60(21)	122.78(28)	123.1(31)	124(3)
∠(C4C4aC4b) / deg	148.00	148.00(30)	148.232(81)	148.206(86)	148.227(84)	148.3(12)	149(2)
∠(C4aC4bC5) / deg	147.64	147.64(30)	147.524(54)	147.509(58)	147.523(55)	148.3(12)	147.0(6)
∠(C4bC5C6) / deg	115.40	115.40(30)	115.308(78)	115.273(89)	115.318(78)	115.9(12)	114.5(7)
∠(C5C6C7) / deg	122.15	122.15(30)	122.17(10)	122.20(11)	122.16(10)	122.6(6)	122.4(3)
∠(C6C7C8) / deg	122.23	122.23(30)	122.109(84)	122.114(85)	122.106(83)	121.2(9)	122.5(4)
∠(C7C8C8a) / deg	115.14	115.14(30)	115.01(16)	115.01(16)	114.99(16)	116.1(18)	115.8(1)
Rovib. Parameters							
c_a / u$^{1/2}$Å			0.248(31)	0.234(36)	0.249(30)		
c_b / u$^{1/2}$Å			0.460(47)	0.50(10)	0.462(47)		
c_c / u$^{1/2}$Å			[0.460(47)]	0.57(10)	[0.462(47)]		
d_a / u$^{1/2}$Å^2			-0.95(17)	-0.88(19)	-0.95(17)		
d_b / u$^{1/2}$Å^2			-4.53(58)	-4.7(1.1)	-4.49(58)		
d_c / u$^{1/2}$Å^2			[-4.53(58)]	-6.1(1.4)	[-4.49(58)]		

Hydrogen atoms		
r(C2-H) / Å	1.0811(10)	1.08109(86)
r(C3-H) / Å	1.0815(10)	1.08152(86)
r(C4-H) / Å	1.0811(10)	1.08112(86)
r(C5-H) / Å	1.0813(10)	1.08132(86)
r(C6-H) / Å	1.0816(10)	1.08161(86)
r(C7-H) / Å	1.0816(10)	1.08158(86)
r(C8-H) / Å	1.0811(10)	1.08107(86)
∠(C1C2H) / deg	118.92(30)	118.96(25)
∠(C2C3H) / deg	118.29(30)	118.32(25)
∠(C3C4H) / deg	120.93(30)	120.92(25)
∠(C4bC5H) / deg	123.52(30)	123.51(26)
∠(C5C6H) / deg	119.16(30)	119.12(25)
∠(C6C7H) / deg	118.64(30)	118.61(26)
∠(C7C8H) / deg	121.45(30)	121.47(25)

[a] Four different rovibrational constants fitted, i.e. $c_b = c_c$ and $d_b = d_c$.
[b] All six rovibrational constants fitted.
[c] All internal coordinates of the hydrogen atoms fitted.
[d] Uncertainties expressed in parentheses in units of the last digit.

Table S2. Molecular structure of 2-cyanobiphenylene, including the *ab initio* calculations, the *ab initio* predicates, the mass-averaged equilibrium structure ($r_m^{(2)}$), the effective structure (r_0) and the substitution structure (r_s). The final equilibrium structure corresponds to the $r_m^{(2)}$ fit with four rovibrational parameters. All internal coordinates of the hydrogen atoms were fixed to the *ab initio* predicates.

	MP2/ cc-pVTZ	Predicate	$r_m^{(2)\,a}$	r_0	r_s
Bond lengths					
r(C1-C2) / Å	1.4196			1.439(43)	1.454(18)
r(C2-C3) / Å	1.3989	1.3967(20)	1.3953(17)	1.394(22)	1.361(26)
r(C3-C4) / Å	1.4087	1.4065(20)	1.4080(15)	1.422(37)	1.419(3)
r(C4-C4a) / Å	1.3753	1.3731(20)	1.3737(15)	1.364(44)	1.295(21)
r(C4a-C8b) / Å	1.4199	1.4199(20)	1.4184(17)	1.422(40)	1.406(4)
r(C8b-C1) / Å	1.3721	1.3699(20)	1.3699(17)	1.357(137)	1.481(10)
r(C4a-C4b) / Å	1.5031	1.5031(20)	1.5019(16)	1.517(65)	1.607(29)
r(C4b-C5) / Å	1.3757	1.3735(20)	1.3708(13)	1.364(48)	1.376(1)
r(C5-C6) / Å	1.4114	1.4092(20)	1.4103(14)	1.425(24)	1.423(4)
r(C6-C7) / Å	1.3937	1.3915(20)	1.3930(15)	1.389(18)	1.384(6)
r(C7-C8) / Å	1.4117	1.4095(20)	1.4128(14)	1.419(19)	1.423(1)
r(C8-C8a) / Å	1.3749	1.3727(20)	1.3725(14)	1.381(39)	1.378(2)
r(C8a-C8b) / Å	1.5042			1.504(146)	1.359(10)
r(C2-C$_\alpha$) / Å	1.4297	1.4334(20)	1.4331(13)	1.436(21)	1.451(9)
r(C$_\alpha$-N) / Å	1.1738	1.1588(20)	1.1552(10)	1.164(26)	1.160(1)
Bond angles					
∠(C1-C2-C3) / deg	122.42			122.5(21)	123.2(6)
∠(C2-C3-C4) / deg	121.59	121.59(30)	121.476(86)	121.5(15)	121.7(3)
∠(C3-C4-C4a) / deg	115.85	115.85(30)	115.901(98)	115.5(16)	112.3(9)
∠(C4-C4a-C8b) / deg	122.41	122.41(30)	122.59(14)	122.8(66)	135.8(22)
∠(C4a-C8b-C1) / deg	122.68	122.68(30)	122.53(18)	123.4(84)	110.8(13)
∠(C4-C4a-C4b) / deg	147.59	147.59(30)	147.76(12)	147.0(22)	143.5(11)
∠(C4a-C4b-C5) / deg	147.53	147.53(30)	147.30(15)	146.9(14)	147.8(2)
∠(C4b-C5-C6) / deg	115.37	115.37(30)	115.50(11)	115.0(13)	115.2(2)
∠(C5-C6-C7) / deg	122.20	122.20(30)	121.966(73)	122.1(12)	122.1(1)
∠(C6-C7-C8) / deg	122.07	122.07(30)	122.064(75)	122.0(10)	122.1(1)
∠(C7-C8-C8a) / deg	115.40	115.40(30)	115.65(13)	116.0(16)	115.7(1)
∠(C$_\alpha$-C2-C3) / deg	118.74	118.74(30)	118.60(11)	119.2(16)	120.0(12)
Rovib. Parameters					
c_a / u$^{1/2}$Å			0.191(43)		
c_b / u$^{1/2}$Å			0.446(64)		
c_c / u$^{1/2}$Å			[0.446(64)]		
d_a / u$^{1/2}$Å^2			-0.57(27)		
d_b / u$^{1/2}$Å^2			-4.84(68)		
d_c / u$^{1/2}$Å^2			[-4.84(68)]		

[a] Four different rovibrational constants fitted, i.e. $c_b = c_c$ and $d_b = d_c$.

Table S3. A selection of carbon-carbon (C-C) bond lengths for partial double bonds (Å) used in the fit of equation S1.

Molecule	Formula (bond)	r_e [a]	MP2 [b]	Offset [c]	Residual [d]
diacetylene	HCC-CCH	1.3727	1.3687	0.0040	-0.0018
methyldiacetylene	CH_3CC-CCH (C_2C_3)	1.3734	1.3676	0.0058	-0.0001
cyanoacetylene	HCC-CN	1.3764	1.3720	0.0044	-0.0013
ethanedinitrile	NC-CN	1.3840	1.3760	0.0080	0.0024
pyrazole	*c*-$C_3H_4N_2$ (C_3C_4)	1.4093	1.4037	0.0056	0.0010
cis-hexene diyne	C_6H_4	1.4210	1.4172	0.0038	-0.0003
pyrrole	*c*-C_4H_5N (C_3C_4)	1.4230	1.4160	0.0070	0.0028
furane	*c*-C_4H_4O	1.4320	1.4255	0.0065	0.0027
phenylacetylene	*c*-C_6H_5CCH (C_1C_7)	1.4322	1.4271	0.0051	0.0013
vinyl cyanide	H_2C=CHCN	1.4315	1.4290	0.0025	-0.0012
benzonitrile	*c*-C_6H_5CN	1.4343	1.4309	0.0034	-0.0003
4-cyanopyridine	*c*-$C_7H_4N_2$ (C_4C_7)	1.4368	1.4304	0.0064	0.0027
tt-difluorobutadiene	$C_4H_4F_2$	1.4502	1.4479	0.0023	-0.0008
hexatriene c	C_6H_8	1.4512	1.4488	0.0024	-0.0006
hexatriene t	C_6H_8	1.4494	1.4464	0.0030	-0.0001
uracil	*c*-$C_4H_4N_2O_2$ (C_4C_5)	1.4539	1.4534	0.0005	-0.0024
cc-difluorobutadiene	$C_4H_4F_2$	1.4505	1.4475	0.0030	-0.0001
methyldiacetylene	CH_3CC-CCH (C_4C_5)	1.4569	1.4552	0.0017	-0.0011
propyne	CH_3CCH	1.4588	1.4583	0.0005	-0.0022
methylcyanide	CH_3CN	1.4586	1.4575	0.0011	-0.0016
oxirane	*c*-C_2H_4O	1.4608	1.4622	-0.0014	-0.0040
glycidol	*c*-$C_3H_6O_2$ (C_2C_3)	1.4612	1.4595	0.0017	-0.0010
ethyl cyanide	C_2H_5CN (CH_2-CN)	1.4639	1.4610	0.0029	0.0003
ethynylcyclohexane	C_8H_{12} (C_1C_7)	1.4633	1.4583	0.0050	0.0023
acrolein	C_3H_4O	1.4700	1.4700	0.0000	-0.0023
fumaric acid	$C_4H_4O_4$ ct	1.4784	1.4760	0.0024	0.0003
aziridine	C_2H_4NH	1.4770	1.4740	0.0030	0.0008
fumaric acid	$C_4H_4O_4$ tt	1.4795	1.4773	0.0022	0.0001
fumaric acid	$C_4H_4O_4$ cc	1.4823	1.4802	0.0021	0.0001
maleic anhydride	*c*-$C_2H_2(CO)_2O$	1.4849	1.4852	-0.0003	-0.0021
diallyldisulfide	$C_6H_{10}S_2$	1.4891	1.4857	0.0034	0.0016
propene	CH_3CH=CH_2	1.4956	1.4952	0.0004	-0.0010
propanal	CH_3CH_2CHO	1.5023	1.5024	-0.0001	-0.0013
cyclopropane	*c*-C_3H_6	1.5030	1.4984	0.0046	0.0033
glycidol	*c*-$C_3H_6O_2$ (C_1C_2)	1.5064	1.5056	0.0008	-0.0003
N-Methylpiperidone	*c*-$C_6H_{11}NO$ (C_2C_3)	1.5102	1.5090	0.0012	0.0002
tropinone	*c*-$C_6H_{11}NO$ (C_1C_2)	1.5140	1.5110	0.0030	0.0021
glycine Ip	$C_2H_5NO_2$	1.5141	1.5131	0.0010	0.0002
glyoxal t	CHO-CHO	1.5145	1.5142	0.0003	-0.0004
dicyanocyclobutene	*c*-$C_4H_4(CN_2)_2$ (C_1C_2)	1.5160	1.5133	0.0027	0.0019

[a] Equilibrium distance, see: Vogt N., Vogt J., *Structure data of free polyatomic molecules*. Springer Nature, Switzerland, 2019, and Hirota E., Kuchitsu K., Steimle T., Vogt J., Vogt N., *Structure data of free polyatomic molecules*, in Kuchitsu K., Vogt N., Tanimoto M. (eds), Landolt-Börnstein, New series: molecules and radicals, vol II/30. Springer, Berlin (and previous editions), 2014. [b] MP2/cc-pVTZ calculation. [c] Offset = (r_e) – MP2/cc-pVTZ. [d] Residual from the fit with Eq. (S1).

Table S4. Atoms-in-molecules (AIM) analysis for 1-cyanobiphenylene.

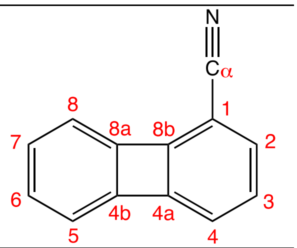

Bond	ρ (a.u.) [a]	ε [b]
N-Cα	0.4885	0.018
C1-Cα	0.2795	0.073
C1-C2	0.2904	0.164
C2-C3	0.3200	0.215
C3-C4	0.2955	0.148
C4-C4a	0.3263	0.213
C4a-C8b	0.3070	0.150
C8b-C1	0.3206	0.224
C4a-C4b	0.2432	0.037
C4b-C8a	0.3070	0.159
C4b-C5	0.3249	0.211
C5-C6	0.2978	0.155
C6-C7	0.3156	0.200
C7-C8	0.2985	0.155
C8-C8a	0.3247	0.208
C8a-C8b	0.2472	0.042

[a]Electronic density in atomic units (e/Å^3).
[b]Bond ellipticity (adimensional).

Table S5. Atoms-in-molecules (AIM) analysis for 2-cyanobiphenylene.

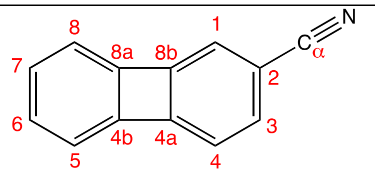

Bond	ρ (a.u.) [a]	ε [b]
N-Cα	0.4880	0.020
C2-Cα	0.2792	0.076
C1-C2	0.2904	0.166
C2-C3	0.3111	0.215
C3-C4	0.3000	0.159
C4-C4a	0.3251	0.206
C4a-C8b	0.3053	0.144
C8b-C1	0.3284	0.222
C4a-C4b	0.2454	0.038
C4b-C8a	0.3074	0.160
C4b-C5	0.3243	0.208
C5-C6	0.2985	0.157
C6-C7	0.3156	0.200
C7-C8	0.2983	0.156
C8-C8a	0.3247	0.210
C8a-C8b	0.2441	0.037

[a] Electronic density in atomic units (e/Å^3).
[b] Bond ellipticity (adimensional).

Table S6. Values for the C≡N equilibrium bond length (Å).

Molecule	MP2/cc-pVTZ	$r_{e.}$	Offset	Ref.[a]
HCC-CN	1.1769	1.1605	0.0164	18
NC-CN	1.1755	1.1578	0.0177	19
c-C_6H_5CN	1.1732	1.1583	0.0149	21
CH_3CN	1.1692	1.1554	0.0138	22
CH_2=CHCN	1.1727	1.1584	0.0143	27
NCNC	1.1738	1.1581	0.0157	28
HCN	1.1668	1.1532	0.0136	29
FCN	1.1712	1.1568	0.0144	30
HOCN	1.1723	1.1579	0.0144	31
ClCN	1.1750	1.1584	0.0166	32

[a] References for the equilibrium structures, see page 15.

Table S7. A comparison of some of the *ab initio* predicates with the substitution structure calculation in Tables S1 and S2.

	1-cyanobiphenylene	
Bond	Predicate	r_s
CN	1.159	1.159(1)
C2C3	1.387	1.389(2)
C4bC5	1.372	1.372(1)
